# Aged Pickle Brine Influences Flavor and Bacteria Characteristics of Pickled Chili Peppers

**DOI:** 10.3390/foods15142564

**Published:** 2026-07-21

**Authors:** Chenshuo Wang, Kaishuang Wei, Zhiji Qin, Zijian Cai, Chenglin Zhu, Zilin Shen, Jiazhuo Gao, Shihong Fang, Zhiyi Fan, Meimei Shi, Ting Li, Weiqin Deng, Luca Laghi

**Affiliations:** 1 College of Pharmacy and Food, Southwest Minzu University, Chengdu 610041, China; 202330827006@stu.swun.edu.cn (C.W.); 202430827078@stu.swun.edu.cn (K.W.); 202430827101@stu.swun.edu.cn (Z.Q.); chenglin.zhu@swun.edu.cn (C.Z.); 2 College of Computer Science and Artificial Intelligence, Southwest Minzu University, Chengdu 610041, China; 202430809135@stu.swun.edu.cn (Z.S.); 202330809144@stu.swun.edu.cn (J.G.); fsh@swun.edu.cn (S.F.); 3 Sichuan Academy of Food and Fermentation Industries Co., Ltd., Chengdu 610041, China; fanzhiyi1992@outlook.com (Z.F.); shimm7280@outlook.com (M.S.); lit.yu@outlook.com (T.L.); dengweiqin77@outlook.com (W.D.); 4 Food Microbiology Key Laboratory of Sichan Province, Chengdu 610041, China; 5 Department of Agricultural and Food Sciences, University of Bologna, 47521 Cesena, Italy; l.laghi@unibo.it

**Keywords:** pickle brine, pickled chili peppers, flavoromic analysis, bacteria communities, correlation analysis, machine learning, multi-omics

## Abstract

Aging duration of the brine is considered one of the core variables determining the fermentation quality of pickled chili peppers. This study systematically analyzed the dynamic changes in volatile and non-volatile metabolites and bacteria community structure in fermented pickled chili peppers fermented with different pickle brines (aged 0, 5, 15, 25, and 50 years). Results indicate that brine age was significantly associated with both the flavor compound composition and bacteria diversity of fermented pickled chili peppers. GC-MS identified 127 volatile compounds, and RF-based exploratory marker analysis identified 10 candidate volatile markers associated with brine aging. ^1^H-NMR analysis screening identified six candidate non-volatile metabolites, whose dynamic changes were associated with bacterial degradation of proteins and carbohydrates. The selected marker panels showed internal discriminative consistency within the current dataset, indicating that brine ageing duration was associated with distinguishable metabolic profiles in fermented pickled chili peppers. α-diversity indices showed both species richness and community diversity shifted dynamically. *Bacillota* dominated the bacterial community, with *Levilactobacillus* as the most abundant genus. Spearman correlation analysis revealed that *Levilactobacillus* was significantly positively correlated with 4-ethyl-2-methoxyphenol, 4-ethylphenol, methylguanidine, and propylene glycol, while negatively correlated with β-Ionone and ethanol. Meanwhile, ethanol was positively associated with *Weissella*, *Oceanobacillus*, and *unclassified_f_Bacillaceae*. These findings provide a theoretical basis for understanding the role of aged brine in pickled chili peppers and support the potential application of multi-omics combined with machine learning to assist in fermented food discrimination. However, it is worth noting that, as the initial metabolic composition of the brines was not characterized, the observed differences should be interpreted as a brine-age-associated composite effect rather than being attributed exclusively to the fermentation process.

## 1. Introduction

As a quintessential example of China’s traditional fermented foods, Sichuan pickles largely owe their distinctive flavor and consistent quality to aged pickle brine, a key element of traditional craftsmanship [1]. Through prolonged use, the brine develops a stable microbial ecosystem rich in beneficial bacterial communities, such as *Lactobacillus* and yeasts, alongside abundant metabolic by-products [2]. Previous studies have suggested that compared to fresh pickle brine, aged pickle brine may shorten fermentation cycles, inhibit undesirable microorganisms, and contribute to the synthesis of complex flavor compounds [3]. However, with the advancement of industrial production, scientifically deciphering the advantageous mechanisms of aged pickle brine and achieving a standardized application have become pivotal issues in the modernization of traditional craftsmanship.

Pickled chili peppers, as a distinctive fermented vegetable product of Sichuan, undergo a fermentation process influenced by raw material characteristics, process parameters, and environmental factors [4,5,6]. Among these factors, the aging duration of the brine is considered one of the core variables determining fermentation quality. Significant differences exist between fresh and reused brine in microbial community structure, metabolic activity, and flavor precursor content [7,8]. Research confirms that the primary flavor compounds in fermented chilies comprise organic acids and free amino acids [9], while key aromatic constituents are concentrated in esters and terpenoid compounds [10]. The formation of flavor substances during fermented chili production is intrinsically linked to microbial metabolism. For example, a study by Liu et al. demonstrated that *Kazachstania* is associated with the synthesis of specific flavor compounds such as 3-methyl-1-butanol [11]. In addition, studies by Yin et al. and by Zhao et al. indicate that *Bacillus, Pediococcus* and *Weissella* species are closely associated with the synthesis of esters such as ethyl salicylate and ethyl lactate [12,13]. These investigations overall suggest a functional linkage between specific microbial groups and flavor compound synthesis. Concurrently, Li et al. employed machine learning integrated with multi-omics techniques to identify the microbial and flavor markers associated with fermentation defects during the stacking stage of sauce-flavor Baijiu production. They discovered that abundance changes in critical bacteria (e.g., *Lactobacillus*) correlate closely with the quality of Baijiu fermentation [14]. However, previous studies mainly focused on binary comparisons (fresh and aged brine) or on single-omics analyses. Consequently, a systematic understanding of how aged pickle brines influence the flavor and bacteria characteristics of pickled chili peppers is still lacking.

Advancements in modern analytical techniques have enabled the integration of multi-omics technologies with artificial intelligence models, offering novel methodologies for studying traditional fermented foods. Previous studies have successfully applied machine learning algorithms, such as RF, to predict the flavor quality of fermented foods [15,16,17]. High-throughput approaches, including 16S rRNA gene sequencing, provide a comprehensive characterization of microbial community structures [18], meanwhile metabolomics enables the systematic profiling of flavor compound dynamics [19]. Furthermore, coupled with machine learning algorithms, such as RF and support vector machine (SVM), these analytical techniques can effectively identify biomarkers and key influencing factors within high-dimensional data [20,21]. Together, these approaches provide a useful analytical framework for exploratory flavor classification and candidate marker discovery in traditional fermentation systems, and may help characterize the complex associations between pickle brine aging and fermentation-related profiles.

In light of these considerations, this study systematically analyzed the dynamic changes in volatile and non-volatile metabolites and bacteria community structure in fermented pickled chili peppers fermented by different pickle brines (aged 0, 5, 15, 25, and 50 years). Specifically, we aim to: (i) characterize the associations between pickle brine aging and the flavor compounds as well as the dynamic governing bacterial community structure in pickled chili peppers; and (ii) identify the key associations between bacterial genera and flavor compounds via 16S rRNA sequencing. These findings provide a theoretical basis for understanding the role of aged brine in pickled chili peppers and support the potential application of multi-omics combined with machine learning to assist in fermented food discrimination.

## 2. Materials and Methods

### 2.1. Sample Preparation

The chili peppers (*Capsicum frutescens* L.) and salt used in this study were purchased from a local Chengdu supermarket. Fresh, undamaged red peppers were selected, rinsed, de-stemmed, and drained. To ensure that brine aging was the only experimental variable, the other fermentation conditions were strictly controlled for all the groups: temperature (20–25 °C), container type (500 mL sterile glass jars), fermentation time (31 days), and final salinity (9% *w*/*v*).

**Figure 1:**
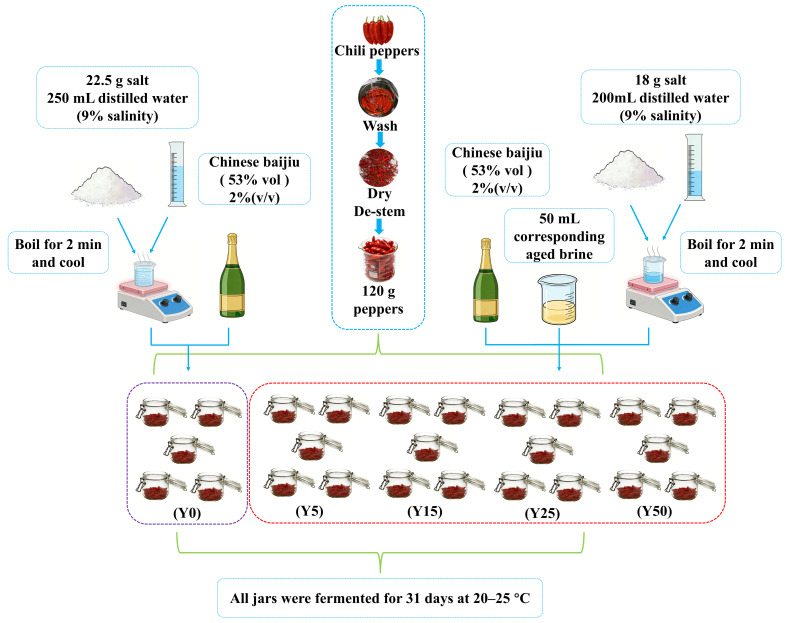
Visual representation of the preparation process of the samples.

As suggested by previous studies, the selection of brine aging time points (0, 5, 15, 25, and 50 years) was justified as follows: 0 years served as fresh brine; 5 and 15 years represent medium-term aging; 25 and 50 years represent long-term to ultra-long-term aging [8,22]. Regarding the sampling procedure, the aged brine samples (Y5, Y15, Y25, and Y50) were obtained from a traditionally operated pickle cellar in Sichuan Province, where the brines had been continuously maintained for the designated periods. Prior to sampling, each brine was gently stirred to ensure homogeneity, and samples were taken from the middle layer using sterile stainless-steel ladles to avoid surface or sediment contamination. The collected samples were immediately transferred into sterile glass bottles, sealed, and transported to the laboratory under refrigerated conditions (4 °C) within 2 h. Reference values for comparable brines are available in Cao et al. [22]. As shown in Figure 1, two experimental designs were implemented, one employing fresh brine (group Y0) and one employing aged brine aged for 5, 15, 25, and 50 years (groups Y5, Y15, Y25, Y50). For the Y0 group, 22.5 g of salt was dissolved in 250 mL of distilled water (9% salinity), boiled for 2 min, cooled, and combined with 120 g of peppers and 2% (*v*/*v*) Chinese baijiu (53% vol) before sealing in 500 mL sterile jars. For the aged pickle brine groups, 18 g of salt was dissolved in 200 mL of distilled water (9% salinity), boiled and cooled, then mixed with 50 mL of corresponding aged pickle brine (fresh-to-aged pickle brine ratio 4:1, *v*/*v*), 120 g of peppers, and 2% (*v*/*v*) Chinese baijiu (53% vol) prior to sealing. The salinity in all experimental groups was uniformly 9% (*w*/*v*). All jars underwent 31-day fermentation at 20–25 °C with five biological replicates per group, and post-fermentation samples were flash-frozen in liquid nitrogen for subsequent analysis.

### 2.2. GC-MS Analysis

Following the methodology of Ma et al. with minor modifications [23], the volatile components of the fermented pickled chili peppers were acquired and analyzed by HS-SPME (solid-phase microextraction fiber, 50/30 μm DVB/CAR/PDMS, Supelco, Bellefonte, PA, USA) and GC-MS (Thermo Fisher Scientific, Waltham, MA, USA) on a TG-WAXMS B column (30 m × 0.25 mm × 0.25 µm), with a Triplus auto-sampler (Thermo Fisher Scientific, Waltham, MA, USA). After mincing each sample, 1.5 g of pickled chili peppers was precisely weighed into a 20 mL headspace bottle and sealed, equilibrated at 60 °C for 30 min with agitation at 250 rpm, and then extracted for 40 min at the same temperature. Desorption was performed at 250 °C for 5 min in splitless mode. Helium (≥99.999% purity) was used as carrier gas in split mode at a constant flow rate of 1.0 mL/min. The oven temperature program was as follows: initial 40 °C held for 3 min, raised at 5 °C/min to 210 °C, and held for 5 min. Mass spectra were acquired in electron ionization mode at 70 eV over the m/z range of 50–550, with an ion source temperature of 280 °C. Compound identification was performed using the NIST 11 library with a match quality score > 800. The classical area normalization method was applied, in which the peak area percentage of each volatile compound was used to represent its relative abundance within the sample.

### 2.3. Relative Odor Activity Value (ROAV) Analysis

Following Sun et al., odor descriptions were obtained with the help of Femaflavour website (https://www.femaflavor.org/flavor-library, accessed on 15 January 2026). The main odor compounds in the fermented pickled chili peppers were found by means of the ROAV, which exhibits the weight of each volatile molecule to the overview of the samples’ flavor [24]. ROAV was calculated in accordance with the following formula:

$${ROAV}_{i}=\frac{C_{i}}{C_{max}}\times \frac{T_{max}}{T_{i}}\times 100$$

Here, C_i_ is the relative content of each flavor compound; T_i_ represents the odor threshold of each flavor compound; C_max_ is the relative content of the compound constituting the largest component of the sample’s overall flavor; and T_max_ denotes the odor threshold of the compound constituting the largest component of the sample’s overall flavor.

### 2.4. ^1^H-NMR Metabolomic Analysis

^1^H-NMR spectroscopy was performed according to Zhu et al. [25] with modifications. Aliquots (1.00 mL) of pickled chili pepper brine were centrifuged (15,000 rpm, 15 min, 4 °C). Each supernatant (0.35 mL) and the same amount of bidistilled water were taken to a new Eppendorf tube. Then, a D_2_O solution of 3-(trimethylsilyl)-propionic-2,2,3,3-d_4_ acid sodium salt (TSP) (10 mmol/L) was added, which could be used as NMR chemical-shift reference. The mixture was buffered at pH 7.00 ± 0.02 by means of phosphate buffer (1 mol/L). Ten microliters of NaN_3_ (2 mmol/L) was added to avoid microbial proliferation. Prior to moving to NMR tubes, all the samples were centrifuged again using the above conditions.

^1^H-NMR spectra were obtained at 298 K by means of an AVANCE III spectrometer (Bruker, Wuhan, China) operating at a frequency of 600.13 MHz. Broad resonances from large molecules were suppressed by a CPMG (Carr-Purcell-Meiboom-Gill) filter (Topspin, Wuhan, China) of 330 ms, which included 400 echoes with a τ of 400 μs and a 180° pulse of 24 μs. Presaturation was used to suppress the heavy water (HOD) residual signal, by employing the cpmgprld sequence, belonging to the standard pulse sequence library. Each spectrum was acquired with an acquisition time of 2.28 s, by summing up 256 transients using 32 K data points over a 7184 Hz spectral window. In order to apply NMR as a quantitative technique [26,27], the recycle delay was set to 5 s, considering the relaxation time of the protons under investigation.

^1^H-NMR spectra phase was manually adjusted by means of Topspin (ver. 4.2), with subsequent adjustment performed through in-house R computational language scripts. After the residual water signal removal, we adjusted the baseline of the spectra by means of peak detection, in accordance with the “rolling ball” principle [28] implemented in the baseline R package [29]. During processing, a line-broadening factor of 0.3 Hz was applied. Differences in water and protein content among samples were taken into consideration by probabilistic quotient normalization (PQN) [30], applied to the entire spectra array. We assigned the signals by means of comparisons of their chemical shift and multiplicity with Chenomx software library (Chenomx Inc., Edmonton, AB, Canada, ver 8.4). Rectangular integration was used to calculate integration of the signals.

### 2.5. Determination of Bacterial Community

Under aseptic conditions, 20 mL samples were taken from pickled brine from different fermentation years in a sterile cryovial, immediately frozen by liquid nitrogen, and stored at −80 °C. Following the manufacturer’s instructions, genomic DNA of the bacterial community was extracted from samples of pickled brine from different fermentation years using the E.Z.N.A.^®^ soil DNA kit (Omega Bio-tek, Norcross, GA, USA). The hypervariable V3-V4 region of the bacterial 16S rRNA gene was amplified with the primers 338F (5′-ACTCCTACGGGAGGCAGCAG-3′), and 806R (5′-GGACTACHVGGGTWTCTAAT-3′) by A T100 Thermal Cycler, BIO-RAD PCR (Thermal, Waltham, MA, USA). Purified amplicons were pooled in equimolar and paired-end sequenced on an Illumina MiSeq PE300/PE250 platform (Illumina, San Diego, CA, USA), according to the standard protocols of Majorbio Bio-Pharm Technology Co., Ltd. (Shanghai, China). Raw reads were demultiplexed, quality-filtered through fastp (Ver. 0.20.0), and merged by FLASH (Ver. 1.2.7). Optimized sequences were processed by DADA2 to obtain the real sequence in formation (Amplicon Sequence Variants, ASVs) in the samples, with the PCR amplification or sequencing errors existing in the optimized sequence removal [31].

### 2.6. Statistical Analysis

#### 2.6.1. Multi-Omics Data Analysis

All experiments were performed with five independent biological replicates (n = 5). Experimental data were analyzed in Excel, and results are expressed as mean ± standard deviation. All graphs were generated with Origin 2021 (OriginLab, Northampton, MA, USA). For differential feature screening across multiple sample groups, *p*-values were obtained using the Kruskal–Wallis non-parametric test, followed by false discovery rate (FDR) correction via the Benjamini–Hochberg method to derive q-values; a threshold of q < 0.05 was used to define statistical significance. When the Kruskal–Wallis test indicated significant differences (q < 0.05), post hoc pairwise comparisons were performed using Dunn’s test with Bonferroni correction to adjust for multiple comparisons. Multivariate analyses, including the RF and principal component analysis (PCA), were implemented in Python (version 3.9). Microbiome data analysis was conducted on the Majorbio cloud platform (https://www.majorbio.com), which included calculation of α-diversity indices (ACE, Chao, Shannon, Sobs, Simpson, and Coverage) and β-diversity analysis based on Bray–Curtis distance, and was visualized using non-metric multidimensional scaling (NMDS) and statistically assessed with analysis of similarities (ANOSIM). Correlations between variables were evaluated by Spearman correlation analysis via the OmicStudio platform (https://www.omicstudio.cn).

#### 2.6.2. Machine Learning Analysis

Machine learning analysis was applied to the GC-MS-derived volatile compound dataset and the ^1^H-NMR-derived non-volatile metabolite dataset. Given the limited biological replication and the high dimensionality of the metabolomic data, the workflow was designed primarily for exploratory candidate marker discovery, with several measures incorporated to reduce overfitting risk, tuning bias, and instability caused by random data partitioning. To support the choice of an RF-based workflow, a full-feature classifier comparison was first conducted separately from marker-panel selection. After label-independent coarse filtering of the raw feature matrix—including removal of all-zero, constant, and low-detection-rate features and replacement of zero values with half of the minimum non-zero value for each feature—11 classifiers were evaluated. These classifiers included logistic regression, linear SVM, radial basis function SVM, k-nearest neighbors (k = 1 and k = 3), shrinkage linear discriminant analysis, Gaussian naive Bayes, XGBoost, Extra-Trees, standard RF, and regularized RF. Model comparison was performed using stratified 5-fold cross-validation and 50-repeat stratified 5-fold cross-validation. Within each cross-validation fold, natural logarithm transformation and Z-score normalization were fitted on the training subset and then applied to the corresponding test subset. Performance was summarized using accuracy, macro-F1, Cohen’s kappa, and Matthews correlation coefficient (Supplementary Figure S1).

Marker panels were then selected in a separate analysis. For each metabolomic dataset, the complete feature matrix was first processed as a whole before statistical screening by removing all-zero, constant, and low-presence features, replacing zero missing values with half of the minimum non-zero value for each feature, and applying natural logarithm transformation and Z-score normalization. In contrast to the full-feature classifier comparison, where ln-transformation and Z-score normalization were applied within cross-validation folds to obtain unbiased performance estimates, the marker-panel construction step preprocessed the complete feature matrix as a whole. This is because the subsequent Kruskal–Wallis screening and RF-based ranking require stable per-feature statistics estimated from all available samples; with only five replicates per brine-age group, further partitioning would reduce per-class sample sizes below the threshold for reliable non-parametric testing and ranking. Features with significant group differences were identified using Kruskal–Wallis tests with Benjamini–Hochberg FDR correction (q < 0.05). The statistically screened features were then ranked by RF Gini importance using fixed RF settings (700 trees, max_depth = 4, and max_features = “sqrt”). Automated hyperparameter optimization was not applied in the RF-ranking step to avoid additional tuning bias under the small sample size.

Based on the full-dataset RF Gini-importance ranking, marker subsets were con-structed by sequentially adding features from the top of the ranking list. Each Top-N subset was evaluated using stratified 5-fold cross-validation, and the subset size with the highest mean cross-validation accuracy was selected as the compact marker panel. When multiple subset sizes reached the same maximum mean accuracy, the smallest subset was retained to avoid unnecessary feature expansion. This procedure yielded a GC-MS Top10 volatile marker panel and a ^1^H-NMR Top6 non-volatile metabolite panel for subsequent internal stability assessment. The Top-N cross-validation results were used to describe internal discrimination within the current dataset.

After the GC-MS Top10 and ^1^H-NMR Top6 marker panels had been selected, in-ternal stability and overfitting-control checks were performed on the fixed panels. These checks examined whether the selected panels remained discriminative after reducing model flexibility and whether the results were sensitive to tree-ensemble randomization, random data partitioning, or random label assignment. These checks were performed after marker selection and were not used to reselect features.

(i) Conservative RF: A conservative RF model with restricted complexity (max_depth = 4, min_samples_leaf = 2, min_samples_split = 5, max_samples = 0.8, and max_features = “sqrt”) was used to reduce model flexibility and assess whether the selected panels retained discriminative performance under a less flexible RF setting.

(ii) Extra-Trees: An Extra-Trees classifier was applied to examine whether the selected panels remained discriminative under a more randomized tree-ensemble splitting strategy, thereby reducing reliance on a single RF splitting mechanism.

(iii) Repeated cross-validation: Stratified 5-fold cross-validation was repeated 50 times to evaluate whether the performance estimates were stable across different random data partitions.

(iv) Permutation test: Selected-marker permutation tests with 1000 random label shuffles were conducted to evaluate whether comparable classification accuracy could arise after disrupting the relationship between the selected markers and the class labels. The empirical *p*-value was calculated as:

$$p\;=\;\frac{\left(1+number\;of\;permuted\;accuracies\geq observed\;accuracy\right)}{\left(1+number\;of\;permutations\right)}$$

Together, these analyses assessed the internal stability of the selected marker panels and limited overinterpretation of classification performance under the current experimental conditions. They did not constitute external validation; therefore, the selected panels should be regarded as exploratory candidate markers.

## 3. Results

### 3.1. GC-MS Analysis

#### 3.1.1. Flavor Characterization

**Figure 2:**
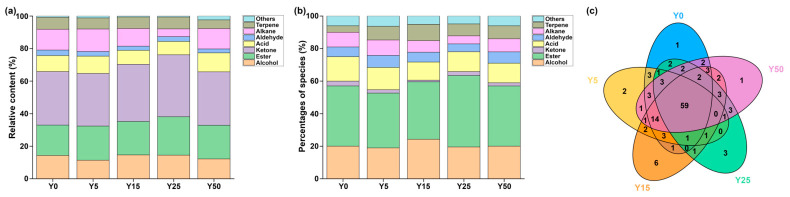
Comparative analysis of volatile compounds in pickled chili peppers fermented with pickle brine from different years. Bar plot of relative contents of volatile compound species in pickled chili peppers during fermentation across pickle brine from different years characterized by GC-MS (**a**). Bar graph of percentages of volatile compound species in pickled chili peppers during fermentation (**b**). Venn diagram (**c**).

GC-MS analysis identified 127 volatile compounds in pickled chili peppers across pickle brine from different years, categorized as alcohols (27), esters (45), ketones (3), acids (17), aldehydes (7), alkanes (9), terpenes (12) and others (2) (as shown in Table S1). The relative content of these compounds differed among groups (Figure 2a). From a compositional perspective, ketones consistently exhibited the highest relative content across all groups. Alcohols, esters, and ketones were the dominant volatile fractions in fermented pickled chili peppers, while the fermentation stage was mainly evident in the concentration of esters, alcohols, acids, and ketones, which was in line with the findings by Liu et al. [32]. A number of volatile compounds equal to 100, 95, 99, 82, and 100 were detected in the Y0, Y5, Y15, Y25, and Y50 groups, respectively, with relative percentages varying among groups (Figure 2b), especially for esters. Figure 2c shows that there were 59 shared volatile compounds in pickled chili peppers fermented with pickle brine from different years. The Y15 group exhibited the highest number of unique volatile compounds, with a gradual increase from Y0 to Y15 followed by a gradual decrease from Y15 to Y50.

#### 3.1.2. GC-MS Combined with Machine Learning Analysis

**Figure 3:**
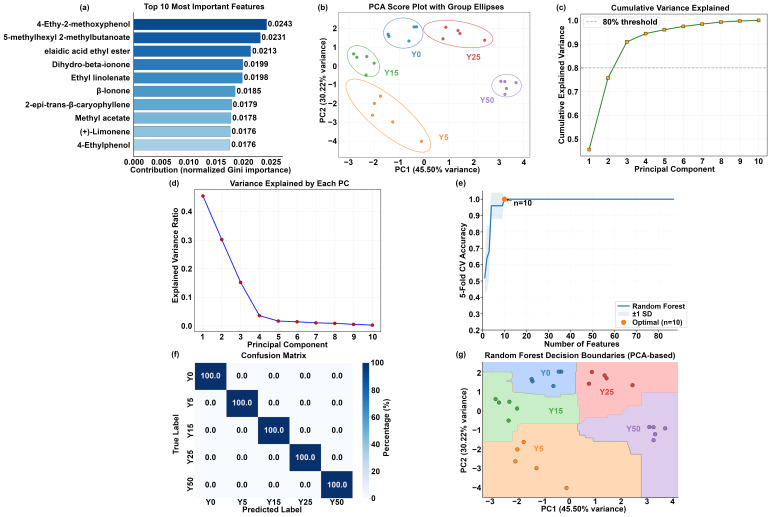
Integrated analysis of volatile organic compound (VOC) marker panel for pickled chili peppers fermented with brine of different ages. Top 10 most important VOC markers identified by RF-based feature ranking, ranked by normalized Gini importance (**a**). PCA score plot with 95% confidence ellipses (**b**). Cumulative variance explained by the principal components (**c**). Variance explained by each individual principal component (**d**). Five-fold cross-validation accuracy of RF models trained on different numbers of top features (**e**). Confusion matrix for the RF classifier using the selected Top10 VOC panel (**f**). RF decision boundaries visualized within the PCA space (**g**).

Based on RF-based feature ranking, the top 10 candidate volatile markers (Figure 3a) identified were 4-ethyl-2-methoxyphenol, 5-methylhexyl 2-methylbutanoate, elaidic acid ethyl ester, dihydro-beta-ionone, ethyl linolenate, β-ionone, 2-epi-trans-β-caryophyllene, methyl acetate, (+)-limonene, and 4-ethylphenol, with phenolic, ester, and terpenoid as the compounds contributing most significantly.

PCA score plots (Figure 3b) revealed clear gradient separation and within-group clustering along the first two principal components, suggesting that brine aging time is associated with the flavor profile of fermented pickled chili peppers. The RF classifier based on the fixed Top10 panel showed high internal cross-validation performance within the current dataset (Figure 3e). The confusion matrix and PCA-space decision-boundary visualization further illustrated the separation pattern among the five groups under the current experimental conditions (Figure 3f,g). Considering that the marker panel was derived from the same dataset, these classification results were interpreted as internal evidence supporting the discriminatory value of the candidate volatile marker panel.

### 3.2. ROAV Analysis

Odor Activity Value serves as an effective criterion for evaluating flavor, as it sheds light on the primary factors of taste detectable by taste buds [33]. Compounds with ROAV ≥ 1 are generally recognized as key flavor compounds, while those with 0.1 ≤ ROAV < 1 also play crucial roles in altering overall flavor profiles [34]. Across all samples, 20 volatile flavor compounds with ROAV ≥ 0.1 were identified (as shown in Table S2). The number of compounds with ROAV ≥ 1 were 4, 2, 2, 3, and 2 for Y0, Y5, Y15, Y25, and Y50, respectively, while those with 0.1 ≤ ROAV < 1 were 7, 9, 8, 13, and 12 for the same samples. Notably, 2-methoxy-3-isobutyl pyrazine emerged as a shared key flavor compound across pickle brine samples from different years, and might thereby play a key role in the flavor characteristic of pickle brine.

Data analysis indicates that 2-methoxy-3-isobutyl pyrazine (exhibiting green bean and green pepper notes) was identified as a core contributor (ROAV = 100) in Y5, Y15, and Y25 group samples, particularly prominent in mid-vintage pickle brine, imparting the characteristic fermented green aroma to pickled chili peppers. 4-ethyl-2-methoxyphenol (contributing smoky and spicy notes) showed significant contribution in the Y50 group (ROAV = 0.24), imparting a distinctive spicy undertone to the samples, which suggested a potential association between longer brine aging and the accumulation of smoky phenolic compounds. (2R,3R)-(-)-2,3-Butanediol was identified as a key flavor compound solely in the Y0 group (ROAV = 100), with no significant contribution detected in samples from other fermentation years, serving as a potential marker for the initial fermentation stage.

### 3.3. ^1^H-NMR Analysis

**Figure 4:**
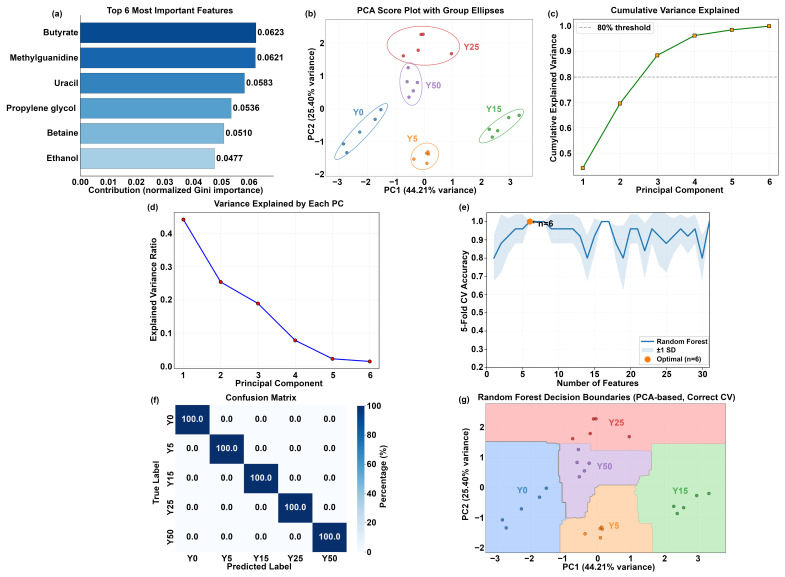
Integrated analysis of discriminant metabolites and marker panel for pickled chili peppers of different brine fermentation ages. Top 6 most important discriminant metabolites identified by RF-based feature ranking, ranked by normalized Gini importance (**a**). PCA score plot with 95% confidence ellipses (**b**). Cumulative variance explained by the principal components (**c**). Variance explained by each individual principal component (**d**). Five-fold cross-validation accuracy of RF models trained on different numbers of top features (**e**). Confusion matrix for the RF classifier using the selected Top6 non-volatile metabolite panel (**f**). RF decision boundaries visualized within the PCA space (**g**).

^1^H-NMR is well-suited for metabolomics investigations due to its minimal sample preparation and high reproducibility, which facilitate real-time analysis of metabolites [35]. The integration values of all identified non-volatile metabolites across the five groups are provided in Supplementary Table S3. Figure 4a lists six candidate non-volatile metabolites ranked by RF importance: butyrate, methylguanidine, uracil, propylene glycol, betaine and ethanol. Figure 4b shows PCA with the first two principal components explaining 44.21% and 25.40% of the total variance, respectively, cumulatively accounting for nearly 70% of the metabolic variation, suggesting distinct metabolic profiles among groups. The variance explanation is further detailed in Figure 4c,d. Figure 4c displays the cumulative variance explained by the principal components, with an 80% threshold line suggesting that approximately three to five components are needed to capture the majority of data variability. Figure 4d confirms that the first few PCs capture the primary sources of metabolic variation, with subsequent components contributing marginally. The five-fold cross-validation accuracy curve (Figure 4e) indicated that the fixed Top6 non-volatile metabolite panel showed high internal discriminative performance within the current dataset. Figure 4f,g show the confusion matrix and decision boundaries, which indicate clear separation among groups within the current dataset. As with the volatile marker panel, these results reflect internal discriminative consistency rather than external validation.

### 3.4. Microbial Communities Analysis

#### 3.4.1. Microbial Taxonomic Composition Analysis

**Figure 5:**
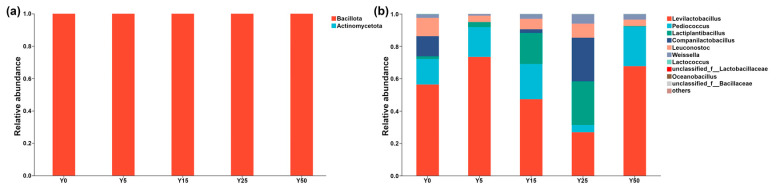
Bacterial community structure dynamic changes in pickled chili peppers fermented with brine of different ages at the phylum (**a**) and genus (**b**) levels.

Microbial community composition analysis was performed on pickled chili peppers samples. As shown in Figure 5a, the top two phyla in relative abundance across all five groups were *Bacillota* and *Actinomycetota*, with *Bacillota* dominating all samples. At the genus level (Figure 5b), *Levilactobacillus*, *Pediococcus*, *Lactiplanibacillus*, *Companilactobacillus*, *Leuconostoc*, *Weissella*, *Lactococcus*, *unclassified_f__Lactobacillaceae*, *Oceanobacillus* and *unclassified_f__Bacillaceae*. *Levilactobacillus* emerged as the most abundant genera across all samples. With increasing pickle brine aging, *Lactiplanibacillus* and *Weissella* progressively increased, even if the former was nearly absent in Y50. These two genera are known to participate in glucose metabolism and organic acid production, which may contribute to flavor development [36]. Other genera, such as *Lactococcus*, *Oceanobacillus*, *unclassified_f__Lactobacillaceae* and *Bacillaceae*, maintained consistently low relative abundances throughout the fermentation process.

#### 3.4.2. Microbial Diversity Analysis

**Figure 6:**
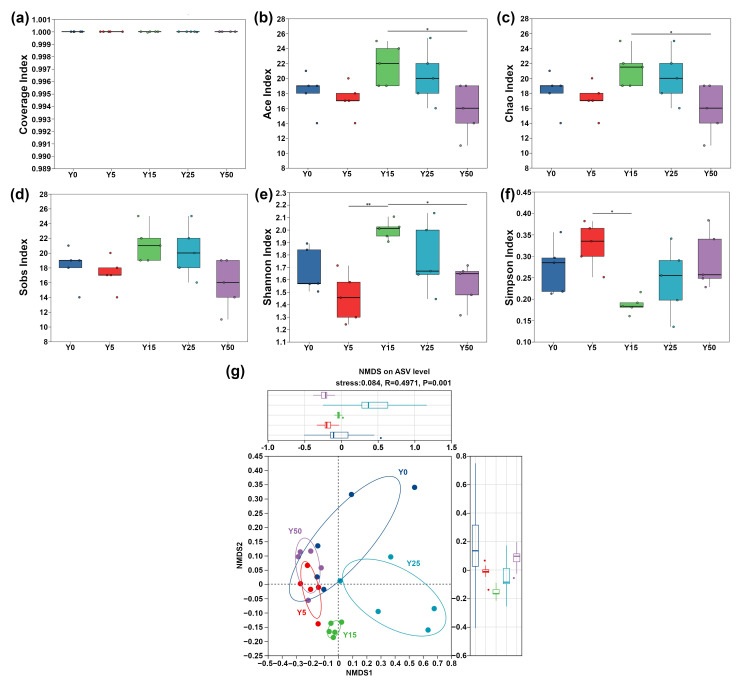
Box plots of *α*-diversity indices of bacterial communities in pickled chili peppers of different brine fermentation ages. Coverage index (**a**); Ace index (**b**); Chao index (**c**); Sobs index (**d**); Shannon index (**e**); Simpson index (**f**). The symbols “*,” “**,” represent significance at *p* < 0.05, *p* < 0.01, and *p* < 0.001, respectively. non-metric multidimensional scaling (NMDS) on ASV level of *β*-diversity in four sample groups (**g**).

This study analyzed the microbial α-diversity and β-diversity of pickled chili peppers samples based on ASV abundance matrices, as shown in Figure 6. Focusing on α-diversity, indices including ACE, Chao1, Sobs, Shannon, and Simpson were calculated to evaluate the richness and diversity of microbial communities [37]. The Coverage index exceeded 0.99 for all samples, indicating sufficient sequencing depth. The Shannon index exhibited a declining trend from Y0 to Y5, peaking in the Y15 group before decreasing significantly (*p* < 0.05) in the Y25 and Y50 groups, reflecting a dynamic process where community diversity first adapts and then simplifies. The Sobs, Chao1, and Ace indices, which characterize species richness, remained relatively stable between the Y5 and Y25 groups but decreased significantly in the Y50 group (*p* < 0.05). Overall, fermentation using less aged brine (Y0) yielded higher bacteria diversity, whereas older brine (Y25 and Y50) resulted in reduced diversity and simplified community structure dominated by a few bacterial taxa. Regarding β-diversity, NMDS revealed that Y15 exhibited greater distance from other groups, suggesting substantial differences in bacteria community structure. This further suggests an association between pickle brine aging and the bacteria community structure of fermented pickled chili peppers.

### 3.5. Correlation Analysis of Microbial Genera, Key Volatile Compounds, and Non-Volatile Compounds

**Figure 7:**
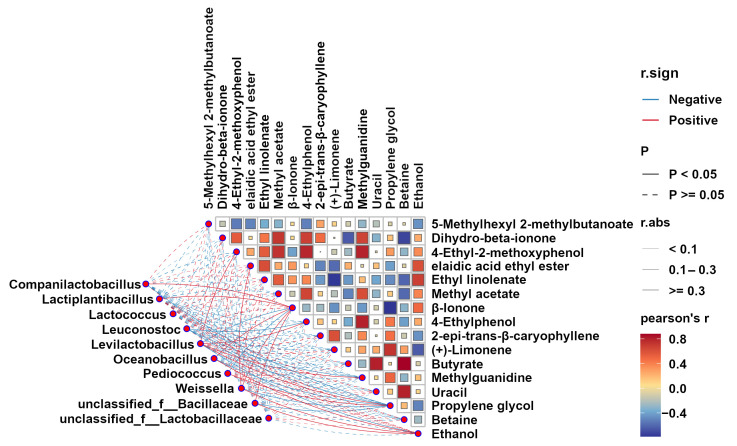
Correlation network heat map of microbial genera and characteristic flavor substances in pickled chili peppers across pickle brine from different years.

As shown in Figure 7, the dominant bacterium *Levilactobacillus* was significantly positively correlated with 4-ethyl-2-methoxyphenol, 4-ethylphenol, methylguanidine and propylene glycol, while being negatively correlated with β-ionone and ethanol. β-ionone was significantly correlated with the highest number of microbial genera, showing positive correlations with *Companilactobacillus*, *Lactiplantibacillus*, *Oceanobacillus*, *Weissella,* and *unclassified_f__Bacillaceae*, while exhibiting negative correlations with *Levilactobacillus* and *Pediococcus*. Ethanol was positively correlated with *Oceanobacillus, Weissella*, and *unclassified_f__Bacillaceae*, but negatively correlated with *Levilactobacillus*, consistent with Wu et al. [36]. Nitrogen-containing compounds such as methylguanidine were primarily positively correlated with *Levilactobacillus* and negatively correlated with *Leuconostoc*.

## 4. Discussion

Sichuan pickled chili peppers, a distinctive fermented vegetable product from Southwest China, owe their characteristic flavor and consistent quality largely to the essence of traditional craftsmanship: aged pickle brine [22]. The aging of the brine is considered as a core factor for fermentation quality, as it develops a stable microbial ecosystem rich in beneficial bacterial communities such as *Lactobacillus* and *yeasts*, alongside abundant metabolic by-products [38]. In the present study, we characterized the flavor profiles and bacteria community dynamics of pickled chili peppers fermented with brine aged from 0 to 50 years using an integrated multi-omics and machine learning approach.

A total of 127 volatile compounds were identified using GC-MS in the pickled chili pepper samples analyzed overall. The volatile figure print of the samples showed significant changes as the fermented pickle brine aging increased. Among them, ten compounds demonstrated significant differences among the five groups, including 4-ethyl-2-methoxyphenol, β-ionone, 5-methylhexyl 2-methylbutanoate, methyl acetate, and others. It is worth noting that Y15, Y25, and Y50 groups exhibited higher abundances of volatile components. Ye et al. also found that aged brine samples contained higher levels of volatile compounds compared to fresh brine, with significantly elevated concentrations of esters and ketones [4]. Esters (e.g., ethyl linolenate, methyl acetate) contribute fruity and sweet notes. Terpenoids (e.g., linalool, β-ionone) impart floral and woody aromas. In addition, phenolics (e.g., 4-ethyl-2-methoxyphenol) contribute smoky and spicy characteristics, and alcohols (e.g., (2R,3R)-(-)-2,3-butanediol) provide creamy and sweet notes.

Alcohols, characterized by their distinct aromas, can be a dominant class of volatile compounds generated through multiple pathways—including the reduction of lipid peroxidation products, carbohydrate degradation, and amino acid catabolism [39,40]. ROAV analysis confirmed that (2R,3R)-(-)-2,3-Butanediol was exclusively detected in the Y0 group (ROAV = 100), serving as a potential marker for the initial fermentation stage. This compound imparts faint sweet and creamy notes and contributed to the flavor profile of Y0. Its accumulation in fresh brine and depletion after Y5 is consistent with Ye et al. [4]. Linalool, a terpene alcohol that imparts a floral and fruity aroma [41], was detected in all groups, with its concentration peaking in Y25 group. This molecule shows a positive association with oxygen-favoring microbes, such as *Metschnikowia pulcherrima* [42] and with *Companilactobacillus versmoldensis* and *Levilactobacillus brevis* [9].

Esters, which have sweet and fruity notes, are formed when carboxylic acids are esterified with alcohols [43]. The sensory detection threshold for esters is lower than that of the corresponding alcohols or acids. The present study found that esters were the most abundant compounds across all five groups, such as 5-methylhexyl 2-methylbutanoate, elaidic acid ethyl ester, ethyl linolenate, and methyl acetate, which aligns with previous reports [9]. The unsaturated fatty acid ester content (e.g., elaidic acid ethyl ester, ethyl linolenate) in the Y25 and Y50 groups was elevated, which may be associated with changes in microbial community composition in aged brine.

Ketones are an important class of volatile flavor compound in pickled chili peppers—with floral, fruity, and woody aromas—and due to their low flavor thresholds, they contribute to the overall flavor profile [44]. Ketones can be generated through the oxidative degradation of unsaturated fatty acids, the cleavage of carotenoids, and microbial secondary metabolism [45]. As a terpene derivative, β-ionone occurs in carotenoid-rich plants, including red peppers, tomatoes, carrots, and teas [46]. β-ionone detection in Y0 group may have resulted from non-enzymatic oxidation of pepper carotenoids and weak initial microbial activity. Its absence in the Y5 and Y15 groups may be associated with the dominance of *Levilactobacillus*, which lacks carotenoid cleavage dioxygenase [47]. Dihydro-β-ionone appeared in the Y5 group, coinciding with the disappearance of β-ionone, which may promote conversion of β-ionone to dihydro-β-ionone. In the Y25 group, the ROAV of dihydro-β-ionone peaked simultaneously with β-ionone, which may suggest balanced metabolic flux between carotenoid cleavage and reduction. In the Y50 group, dihydro-β-ionone remained relatively high while β-ionone was disappeared.

Non-volatile metabolites are important flavor contributors and functional components in fermented chili peppers, directly influencing taste characteristics (e.g., sourness, umami, sweetness) and serving as precursors of volatile compounds [48]. In this study, the dynamic changes in non-volatile metabolites were systematically analyzed by ^1^H-NMR, which identified butyrate, methylguanidine, uracil, propylene glycol, betaine, and ethanol as key discriminant metabolites.

Butyrate may be derived from the metabolic activity of strict anaerobes such as *Clostridia*. Its production can occur via two pathways: one involving phosphotransbutyrylase and butyrate kinase, and the other using butyryl-CoA: acetate-CoA transferase [49]. The peak butyrate accumulation in the Y15 group coincided with the highest bacterial diversity, which may reflect an enrichment of strict anaerobes after 15 years of aging, potentially associated with oxygen depletion and reduced redox potential. The decline in butyrate from Y25 to Y50, while remaining at baseline levels, may suggest the stabilization of the ecosystem function. However, as pre-fermentation butyrate levels were not measured, we cannot rule out the possibility that part of this butyrate was already present in the aged brine prior to fermentation. Ethanol, which can be produced by yeasts and heterofermentative lactic acid bacteria (LAB), showed fluctuating levels across groups, peaking in Y15 and reaching its lowest in Y50. Chinese baijiu was uniformly added to all groups, providing an identical initial ethanol baseline; thus, post-fermentation differences primarily reflect net microbial production and consumption. The Y15 peak likely results from enhanced heterofermentative activity, particularly from genera such as *Weissella* and *Oceanobacillus*, which positively correlated with ethanol [50]. The decline in Y50 may involve reduced heterofermenter abundance, esterification consumption, and possible oxidation, consistent with the negative correlation between ethanol and the dominant genus *Levilactobacillus*. Nevertheless, as the initial composition of the 50-year brine was not characterized, the direct contribution of the brine itself cannot be excluded; therefore, the observed differences represent a brine-age-associated composite effect.

The fermentation of vegetable material is often accompanied by complex dynamic successions of bacteria communities, which shape the final aromatic profile of the products [51]. In this study, bacterial diversity exhibited a dynamic pattern where an initial decrease was followed by an increase, and then by a subsequent decrease. Several reasons may explain this diversity variation in older brines: (i) oxygen depletion over prolonged fermentation creates selective pressure favoring anaerobes; (ii) decreased redox potential favors specific taxa such as *Levilactobacillus*; (iii) accumulation of organic acids and bacteriocins inhibits sensitive species; and (iv) long-term domestication leads to dominance by a few metabolically specialized populations. At the phylum level, *Bacillota* became dominant along fermentation, comprising nearly the entire microbial community, consistently with previous findings [52]. At the genus level, *Levilactobacillus*, *Weissella*, *Lactiplantibacillus*, *Companilactobacillus* and *Pediococcus* exhibited succession patterns across five groups and were identified as the principal genera, in agreement with Xu et al. [53]. In contrast to previous studies, *Levilactobacillus* maintained the highest relative abundance across all groups, consistently with Liang et al. [54], though its dominance exhibited dynamic changes with increasing brine age: it increased from Y0 group to Y5 group, then it gradually decreased from Y5 group to Y25 group, and remained dominant in the Y50 group. The relative abundances of *Weissella* and *Lactiplantibacillus* progressively increased from Y5 group to Y25 group but nearly disappeared in the Y50 group. As a heterofermentative lactic acid bacterium, Weissella metabolizes via the HMP pathway to produce lactic acid, CO_2_, ethanol and acetic acid, wherein CO_2_ enhances carotenoid solubility while microaerophilic conditions promote ester synthesis (e.g., ethyl acetate) [55].

Bacterial composition is associated with the flavor profile of fermented products, with LAB and other functional microbes playing roles in flavor development through metabolic activities such as ester synthesis, alcohol production, and organic acid metabolism [56]. *Weissella* is a heterolactic fermentative bacterium known to produce ethanol and synthesize esters under certain conditions [57]. In the current study, *Weissella* showed significant positive correlation with ethanol and ethyl elaidate, which is consistent with its reported association with flavor formation [58,59]. *Levilactobacillus* participates in carbohydrate decomposition and polyol production during fermentation [60]. Its positive correlation with propylene glycol could be linked to active glycolysis and glycerol metabolism. Meanwhile, *Levilactobacillus* was significantly positively correlated with 4-ethyl-2-methoxyphenol and 4-ethylphenol, which are derived from phenolic acid decarboxylation and reduction metabolism [61].

A limitation of this study is that the initial metabolic composition of the aged brines was not characterized. Therefore, some of the detected metabolites may have originated from the brine itself rather than being generated exclusively through fermentation. Although strict experimental controls were applied, the direct contribution of initial brine composition to the observed differences cannot be fully excluded. Accordingly, the observed inter-group differences should be interpreted as a brine-age-associated composite effect rather than a purely fermentation-driven process, and the reported correlations remain associative rather than causal.

## 5. Conclusions

In this study, pickled chili peppers fermented with pickle brine aged for different periods (0, 5, 15, 25, and 50 years) were used as research materials, using GC-MS, ^1^H-NMR and 16S rRNA amplicon sequencing. The results demonstrated that brine aging duration was significantly correlated with the overall metabolic profiles and microbial community composition of pickled chili peppers, with phenolic, ester, terpene, and organic acid compounds serving as the main discriminatory markers. Microbial analysis showed that *Bacillota* was the dominant phylum across all samples, with *Levilactobacillus* as the most abundant genus. Spearman correlation analysis showed that *Levilactobacillus* was mainly positively correlated with phenols and nitrogen-containing metabolites, while *Companilactobacillus*, *Weissella* and *Oceanobacillus* were strongly associated with terpenoids and ethanol. The RF-based marker-panel analysis showed that the selected volatile and non-volatile compounds had strong internal discriminative ability for differentiating samples fermented with brines of different ages within the current dataset. These findings suggest associations between bacterial taxa and flavor compounds of aged pickle brine, provide a theoretical basis for improving the flavor quality and standardized production of traditional Sichuan pickled chili peppers, and indicate the potential application of multi-omics combined with machine learning in identifying fermentation age markers.

However, it should be noted that the final metabolic composition of fermented pickled chili peppers may originate from three sources: the chili pepper matrix, the brine liquid, and microbial fermentation activities. Since the initial composition of the aged brines was not characterized in this study, we cannot distinguish between metabolites derived from the brine itself and those generated during fermentation. Therefore, the observed differences across groups should be interpreted as a composite effect associated with brine aging, rather than being attributed exclusively to the fermentation process.

## Data Availability

The original contributions presented in this study are included in the article/Supplementary Material. Further inquiries can be directed to the corresponding author.

## Supplementary Materials

The following supporting information can be downloaded at: https://www.mdpi.com/article/10.3390/foods15142564/s1, Figure S1. Full-feature algorithm comparison for GC-MS and ^1^H-NMR metabolomic datasets. Classification performance of different algorithms using all samples and the complete GC-MS volatile compound feature matrix (a,b). Classification performance of different algorithms using all samples and the complete ^1^H-NMR non-volatile metabolite feature matrix (c,d). Supplementary Table S1. Relative content of volatile compounds in pickled chili peppers fermented with pickle brine aged for 0, 5, 15, 25, and 50 years (groups Y0, Y5, Y15, Y25, and Y50) characterized by GC-MS (Mean ± SD). Supplementary Table S2. Aroma characteristics and ROAVs of key volatile compounds in different treatment groups. Supplementary Table S3. ^1^H-NMR-derived concentrations (mmol/L, mean ± SD) of non-volatile metabolites in pickled chili peppers fermented with brine aged for 0, 5, 15, 25, and 50 years (n = 5). Supplementary Methods S1. Mathematical derivations of Gini impurity and feature importance.
