# Supplementary material for: Aged Pickle Brine Influences Flavor and Bacteria Characteristics of Pickled Chili Peppers
Source: Foods. 2026 Jul 21;15(14):2564. doi: 10.3390/foods15142564 (PMC13408934; doi:10.3390/foods15142564)
Supplement: Supplementary file 1 [file foods-15-02564-s001.zip › foods-4400134-supplementary.pdf]

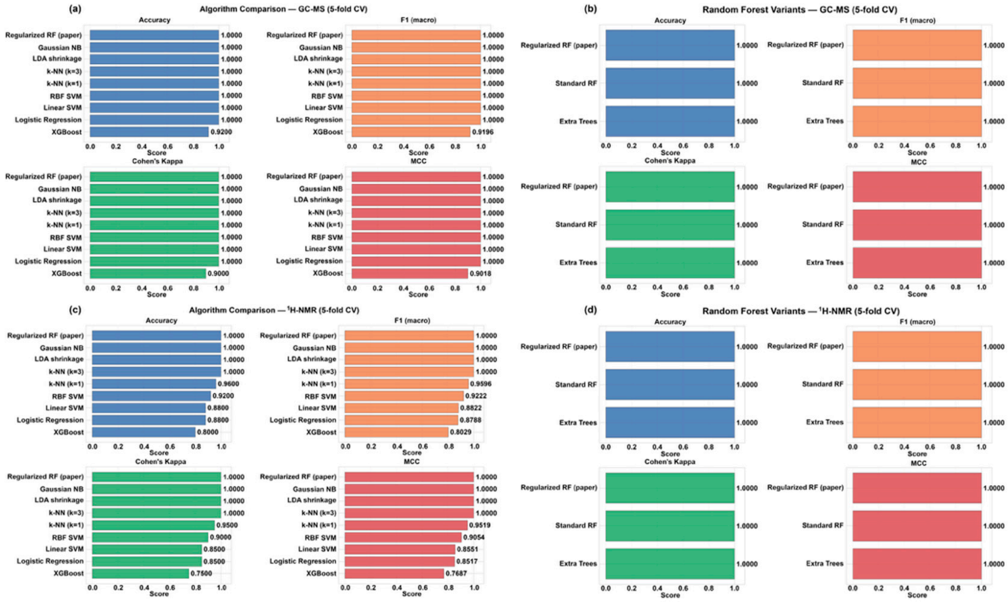

Figure S1. Full-feature algorithm comparison for GC-MS and ^{1}H-NMR metabolomic datasets. Classification performance of different algorithms using all samples and the complete GC-MS volatile compound feature matrix (a)(b). Classification performance of different algorithms using all samples and the complete ^{1}H-NMR non-volatile metabolite feature matrix(c)(d).

Supplementary Methods S1. Mathematical derivations of Gini impurity and feature importance

The Gini impurity of a node D is defined as:

$$\mathrm{Gini}(D)=1-\sum_{k=1}^{k} p_k^2$$

where K is the number of classes (here K = 5) and p_k is the proportion of samples belonging to class k in node D.

The reduction in impurity $\Delta\mathrm{Gini}(D, j)$ brought by splitting node D using feature j is:

$$\Delta\mathrm{Gini}(D,j)=\mathrm{Gini}(D)-\sum_{v\in \mathrm{Split}_j}\frac{|D|}{|D_v|}\mathrm{Gini}(D_v)$$

where Split_j denotes the set of child nodes generated by splitting on feature j, D_v is the v-th child node, and $|D_v|$ and $|D|$ are the sample sizes of the child and parent nodes, respectively.

The final importance Importance(j) of feature j is calculated as:

$$\mathrm{Importance}(j)=\frac{1}{T}\sum_{t=1}^{T}\sum_{m\in M_t(j)}\Delta\mathrm{Gini}(m,j)$$

where T is the total number of trees in the random forest, and $M_{t(j)}$ is the set of nodes in tree t that use feature j for splitting.

Supplementary Table S1. Relative content of volatile compounds in pickled chili peppers fermented with pickle brine aged for 0, 5, 15, 25, and 50 years (groups Y0, Y5, Y15, Y25, and Y50) characterized by GC-MS (Mean ± SD)

Compounds	CAS	Formula	RT (s)	Groups				
				Y0	Y5	Y15	Y25	Y50
**Alcohol**								
Methyl Alcohol	67-56-1	CH_4O	5.35	$1.42\times10^{-2}\pm8.89\times10^{-3}{}^{a}$	$7.50\times10^{-3}\pm4.74\times10^{-3}{}^{a}$	$7.67\times10^{-3}\pm4.73\times10^{-3}{}^{a}$	$1.45\times10^{-2}\pm3.07\times10^{-3}{}^{a}$	$1.05\times10^{-2}\pm9.77\times10^{-3}{}^{a}$
Ethanol	64-17-5	C_2H_6O	5.94	$4.52\times10^{-2}\pm5.26\times10^{-2}{}^{a}$	$3.82\times10^{-2}\pm2.74\times10^{-2}{}^{a}$	$5.83\times10^{-2}\pm6.70\times10^{-2}{}^{a}$	$3.02\times10^{-2}\pm1.35\times10^{-2}{}^{a}$	$2.21\times10^{-2}\pm9.43\times10^{-3}{}^{a}$
3-Pentanol, 2-methyl-	565-67-3	$C_6H_{14}O$	12.40	$8.81\times10^{-4}\pm2.06\times10^{-4}{}^{a}$	$4.50\times10^{-4}\pm1.17\times10^{-4}{}^{b}$	$9.94\times10^{-4}\pm4.35\times10^{-4}{}^{a}$	$9.08\times10^{-4}\pm9.05\times10^{-5}{}^{a}$	$7.87\times10^{-4}\pm2.44\times10^{-4}{}^{ab}$
isoamylol	123-51-3	$C_5H_{12}O$	14.35	$6.42\times10^{-3}\pm3.47\times10^{-3}{}^{bc}$	$6.34\times10^{-3}\pm3.46\times10^{-3}{}^{c}$	$9.41\times10^{-3}\pm3.39\times10^{-3}{}^{bc}$	$3.09\times10^{-2}\pm1.29\times10^{-2}{}^{a}$	$1.16\times10^{-2}\pm2.40\times10^{-3}{}^{b}$
1-Pentanol	71-41-0	$C_5H_{12}O$	16.08	$1.04\times10^{-3}\pm2.94\times10^{-4}{}^{a}$	$1.02\times10^{-3}\pm4.19\times10^{-4}{}^{a}$	$6.86\times10^{-4}\pm9.22\times10^{-5}{}^{b}$	ND	$9.21\times10^{-4}\pm2.54\times10^{-4}{}^{a}$
1-Pentanol, 4-methyl-	626-89-1	$C_6H_{14}O$	18.85	$1.99\times10^{-2}\pm1.11\times10^{-2}{}^{a}$	$1.76\times10^{-2}\pm4.41\times10^{-3}{}^{a}$	$1.76\times10^{-2}\pm2.60\times10^{-3}{}^{a}$	$2.61\times10^{-2}\pm3.59\times10^{-3}{}^{a}$	$2.00\times10^{-2}\pm8.56\times10^{-3}{}^{a}$
1-Hexanol	111-27-3	$C_6H_{14}O$	20.77	$3.83\times10^{-3}\pm6.47\times10^{-4}{}^{a}$	$3.98\times10^{-3}\pm1.20\times10^{-3}{}^{a}$	$2.90\times10^{-3}\pm7.18\times10^{-4}{}^{b}$	ND	$3.23\times10^{-3}\pm8.52\times10^{-4}{}^{ab}$
(2R)-octan-2-ol	5978-70-1	$C_8H_{18}O$	24.10	$3.60\times10^{-2}\pm1.49\times10^{-2}{}^{a}$	$2.55\times10^{-2}\pm1.74\times10^{-2}{}^{a}$	$2.02\times10^{-2}\pm2.03\times10^{-3}{}^{a}$	$3.07\times10^{-2}\pm6.89\times10^{-3}{}^{a}$	$2.27\times10^{-2}\pm6.77\times10^{-3}{}^{a}$
3,5-Dimethyl-2-octanol	19781-09-0	$C_{10}H_{22}O$	29.86	$2.25\times10^{-3}\pm5.68\times10^{-4}{}^{a}$	ND	$1.65\times10^{-3}\pm7.92\times10^{-4}{}^{ab}$	$1.84\times10^{-3}\pm1.18\times10^{-4}{}^{ab}$	$1.34\times10^{-3}\pm5.24\times10^{-4}{}^{b}$
Linalool	78-70-6	$C_{10}H_{18}O$	30.36	$3.47\times10^{-2}\pm9.76\times10^{-3}{}^{a}$	$3.06\times10^{-2}\pm9.68\times10^{-3}{}^{a}$	$2.44\times10^{-2}\pm2.55\times10^{-3}{}^{a}$	$3.84\times10^{-2}\pm5.63\times10^{-3}{}^{a}$	$3.14\times10^{-2}\pm5.85\times10^{-3}{}^{a}$
1-Octanol	111-87-5	$C_8H_{18}O$	30.79	$2.42\times10^{-3}\pm6.74\times10^{-4}{}^{a}$	ND	$2.41\times10^{-3}\pm4.09\times10^{-4}{}^{a}$	ND	$1.37\times10^{-3}\pm1.64\times10^{-4}{}^{c}$
Benzyl alcohol	100-51-6	C_7H_8O	44.84	$4.32\times10^{-3}\pm1.47\times10^{-3}{}^{a}$	$3.82\times10^{-3}\pm1.56\times10^{-3}{}^{a}$	$3.46\times10^{-3}\pm5.19\times10^{-4}{}^{a}$	$5.39\times10^{-3}\pm6.37\times10^{-4}{}^{a}$	$4.10\times10^{-3}\pm1.16\times10^{-3}{}^{a}$
α-Ionol	25312-34-9	$C_{13}H_{22}O$	45.51	$8.54\times10^{-4}\pm2.33\times10^{-4}{}^{a}$	$7.41\times10^{-4}\pm1.35\times10^{-4}{}^{ab}$	$6.73\times10^{-4}\pm7.01\times10^{-5}{}^{b}$	ND	$9.28\times10^{-4}\pm1.73\times10^{-4}{}^{a}$
Phenylethyl Alcohol	60-12-8	$C_8H_{10}O$	46.19	$9.71\times10^{-3}\pm8.82\times10^{-3}{}^{ab}$	$5.87\times10^{-3}\pm2.26\times10^{-3}{}^{b}$	$1.13\times10^{-2}\pm1.06\times10^{-2}{}^{ab}$	$2.45\times10^{-2}\pm2.99\times10^{-3}{}^{a}$	$1.43\times10^{-2}\pm5.42\times10^{-3}{}^{ab}$
dihydro-beta-ionol	3293-47-8	$C_{13}H_{24}O$	47.92	$5.67\times10^{-4}\pm1.90\times10^{-4}{}^{a}$	$6.18\times10^{-4}\pm2.12\times10^{-4}{}^{a}$	$6.05\times10^{-4}\pm1.14\times10^{-4}{}^{a}$	ND	ND
(±)-trans-Nerolidol	40716-66-3	$C_{15}H_{26}O$	49.41	$1.52\times10^{-2}\pm5.92\times10^{-3}{}^{a}$	$1.48\times10^{-2}\pm9.35\times10^{-3}{}^{a}$	$1.57\times10^{-2}\pm4.93\times10^{-3}{}^{a}$	$1.36\times10^{-2}\pm1.94\times10^{-3}{}^{a}$	$1.78\times10^{-2}\pm1.07\times10^{-2}{}^{a}$
(Z)-linalool oxide (furanoid)	5989-33-3	$C_{10}H_{18}O_2$	25.17	$7.75\times10^{-4}\pm2.71\times10^{-4}{}^{c}$	$1.07\times10^{-3}\pm3.45\times10^{-4}{}^{c}$	$2.56\times10^{-3}\pm6.21\times10^{-4}{}^{b}$	$2.48\times10^{-3}\pm2.00\times10^{-4}{}^{b}$	$3.27\times10^{-3}\pm7.80\times10^{-4}{}^{a}$

α-terpineol	98-55-5	$C_{10}H_{18}O$	37.10	$6.75\times10^{-3}\pm2.38\times10^{-3a}$	$6.93\times10^{-3}\pm2.69\times10^{-3a}$	$8.15\times10^{-3}\pm3.05\times10^{-3a}$	ND	$8.65\times10^{-3}\pm1.80\times10^{-3a}$
nerol	106-25-2	$C_{10}H_{18}O$	41.60	$4.14\times10^{-4}\pm9.35\times10^{-5b}$	$9.14\times10^{-4}\pm2.42\times10^{-4a}$	$6.84\times10^{-4}\pm2.16\times10^{-4ab}$	ND	$6.48\times10^{-4}\pm1.37\times10^{-4ab}$
(2R,3R)-(-)-2,3-Butanediol	24347-58-8	$C_4H_{10}O_2$	31.88	$6.80\times10^{-3}\pm4.42\times10^{-3a}$	ND	ND	ND	ND
(S)-(+)-2-Heptanol	6033-23-4	$C_7H_{16}O$	19.23	ND	$1.23\times10^{-3}\pm5.08\times10^{-4a}$	ND	ND	ND
isobutanol	78-83-1	$C_4H_{10}O$	10.28	ND	ND	$7.69\times10^{-4}\pm2.30\times10^{-4c}$	$2.85\times10^{-3}\pm6.60\times10^{-4a}$	$1.85\times10^{-3}\pm3.02\times10^{-4b}$
Propylene Glycol	57-55-6	$C_3H_8O_2$	32.57	ND	ND	$1.37\times10^{-2}\pm7.70\times10^{-3a}$	$6.58\times10^{-3}\pm2.94\times10^{-3b}$	$7.89\times10^{-3}\pm2.97\times10^{-3ab}$
(R)-Citronellol	1117-61-9	$C_{10}H_{20}O$	40.19	ND	ND	$3.97\times10^{-3}\pm1.07\times10^{-3a}$	ND	ND
(±)-2-Butanol	78-92-2	$C_4H_{10}O$	8.05	ND	ND	$1.03\times10^{-3}\pm1.77\times10^{-4b}$	$4.37\times10^{-3}\pm2.73\times10^{-3a}$	ND
1-Propanol	71-23-8	C_3H_8O	8.44	ND	ND	$1.59\times10^{-2}\pm7.21\times10^{-3a}$	ND	ND
(-)-Terpinen-4-ol	20126-76-5	$C_{10}H_{18}O$	32.73	ND	ND	ND	$3.23\times10^{-3}\pm6.82\times10^{-4a}$	ND
**Ester**								
Ethyl Acetate	141-78-6	$C_4H_8O_2$	5.22	$3.05\times10^{-2}\pm1.21\times10^{-2b}$	$1.87\times10^{-2}\pm1.46\times10^{-2b}$	$3.43\times10^{-2}\pm1.11\times10^{-2ab}$	$4.80\times10^{-2}\pm1.44\times10^{-2a}$	$4.93\times10^{-2}\pm9.53\times10^{-3a}$
Ethyl butyrate	105-54-4	$C_6H_{12}O_2$	8.41	$1.20\times10^{-3}\pm5.35\times10^{-4ab}$	$6.64\times10^{-4}\pm2.75\times10^{-4b}$	ND	ND	$1.92\times10^{-3}\pm6.59\times10^{-4a}$
Ethyl 2-methylbutyrate	7452-79-1	$C_7H_{14}O_2$	8.87	$1.24\times10^{-3}\pm5.21\times10^{-4b}$	$1.41\times10^{-3}\pm4.62\times10^{-4b}$	$3.48\times10^{-3}\pm1.20\times10^{-3a}$	$3.28\times10^{-3}\pm1.65\times10^{-3a}$	$2.08\times10^{-3}\pm9.42\times10^{-4ab}$
ethyl isovalerate	108-64-5	$C_7H_{14}O_2$	9.33	$8.10\times10^{-4}\pm3.39\times10^{-4b}$	$7.77\times10^{-4}\pm3.40\times10^{-4b}$	$1.27\times10^{-3}\pm4.66\times10^{-4ab}$	$1.98\times10^{-3}\pm5.47\times10^{-4a}$	$1.63\times10^{-3}\pm1.38\times10^{-4a}$
methyl hexanoate	106-70-7	$C_7H_{14}O_2$	13.63	$7.31\times10^{-4}\pm5.94\times10^{-4b}$	$1.30\times10^{-3}\pm4.48\times10^{-4ab}$	ND	$1.17\times10^{-3}\pm2.11\times10^{-4ab}$	$1.64\times10^{-3}\pm4.83\times10^{-4a}$
ethyl 4-methylpentanoate	25415-67-2	$C_8H_{16}O_2$	13.76	$4.49\times10^{-3}\pm6.04\times10^{-4b}$	$1.14\times10^{-3}\pm7.49\times10^{-4c}$	$3.35\times10^{-3}\pm2.09\times10^{-3b}$	$6.55\times10^{-3}\pm5.29\times10^{-4a}$	$5.19\times10^{-3}\pm2.13\times10^{-3a}$
ethyl hexanoate	123-66-0	$C_8H_{16}O_2$	15.44	$5.08\times10^{-2}\pm2.70\times10^{-2a}$	$6.23\times10^{-2}\pm1.88\times10^{-2a}$	$8.02\times10^{-2}\pm2.97\times10^{-2a}$	$8.36\times10^{-2}\pm1.75\times10^{-2a}$	$5.70\times10^{-2}\pm2.61\times10^{-2a}$
hexyl acetate	142-92-7	$C_8H_{16}O_2$	17.12	$1.17\times10^{-3}\pm6.42\times10^{-4a}$	$1.40\times10^{-3}\pm4.19\times10^{-4a}$	$1.10\times10^{-3}\pm3.94\times10^{-4a}$	$1.40\times10^{-3}\pm7.41\times10^{-4a}$	$1.64\times10^{-3}\pm5.95\times10^{-4a}$
Propanoic acid, 2-hydroxy-, ethyl ester, (L)-	687-47-8	$C_5H_{10}O_3$	20.40	$4.34\times10^{-3}\pm1.76\times10^{-3ab}$	$4.29\times10^{-3}\pm1.60\times10^{-3ab}$	$6.43\times10^{-3}\pm1.23\times10^{-3a}$	ND	$7.96\times10^{-3}\pm2.56\times10^{-3a}$
4-Methylpentyl	35852-40-5	$C_{11}H_{22}O_2$	22.20	$3.56\times10^{-2}\pm2.33\times10^{-2a}$	$3.15\times10^{-2}\pm1.97\times10^{-2a}$	$2.87\times10^{-2}\pm1.80\times10^{-2a}$	$3.56\times10^{-2}\pm8.69\times10^{-3a}$	$3.03\times10^{-2}\pm1.13\times10^{-2a}$

2-methylbutanoate								
4-methyl pentyl isovalerate	850309-45-4	$C_{11}H_{22}O_2$	23.16	$3.41\times10^{-2}\pm1.34\times10^{-2}{}^{ab}$	$6.05\times10^{-2}\pm4.98\times10^{-2}{}^{a}$	$2.96\times10^{-2}\pm2.51\times10^{-2}{}^{b}$	$4.27\times10^{-2}\pm4.79\times10^{-3}{}^{ab}$	$1.90\times10^{-2}\pm1.13\times10^{-2}{}^{b}$
Ethyl caprylate	106-32-1	$C_{10}H_{20}O_2$	24.87	$4.85\times10^{-3}\pm1.13\times10^{-3}{}^{c}$	$8.37\times10^{-3}\pm2.85\times10^{-3}{}^{bc}$	$8.70\times10^{-3}\pm3.32\times10^{-3}{}^{bc}$	$1.47\times10^{-2}\pm2.89\times10^{-3}{}^{a}$	$8.38\times10^{-3}\pm2.28\times10^{-3}{}^{b}$
ethyl nonanoate	123-29-5	$C_{11}H_{22}O_2$	27.55	$1.00\times10^{-2}\pm2.30\times10^{-3}{}^{b}$	$1.62\times10^{-2}\pm5.63\times10^{-3}{}^{ab}$	$1.29\times10^{-2}\pm6.31\times10^{-3}{}^{ab}$	$2.10\times10^{-2}\pm3.44\times10^{-3}{}^{a}$	$1.79\times10^{-2}\pm5.45\times10^{-3}{}^{ab}$
5-Methylhexyl 2-methylbutanoate	117421-30-4	$C_{12}H_{24}$	27.72	$7.50\times10^{-3}\pm3.07\times10^{-3}{}^{a}$	$4.92\times10^{-3}\pm3.72\times10^{-3}{}^{a}$	$2.94\times10^{-3}\pm2.79\times10^{-3}{}^{a}$	$4.18\times10^{-3}\pm1.91\times10^{-3}{}^{a}$	ND
5-Methylhexyl 3-methylbutanoate	1215127-79-9	$C_{12}H_{24}O_2$	28.63	$2.85\times10^{-3}\pm1.03\times10^{-3}{}^{ab}$	$3.96\times10^{-3}\pm1.88\times10^{-3}{}^{a}$	$2.34\times10^{-3}\pm1.13\times10^{-3}{}^{ab}$	ND	$1.43\times10^{-3}\pm1.58\times10^{-4}{}^{b}$
4-Methylpentyl 4-methylpentanoate	35852-42-7	$C_{12}H_{24}O_2$	28.95	$2.70\times10^{-2}\pm2.45\times10^{-2}{}^{a}$	$3.82\times10^{-2}\pm2.62\times10^{-2}{}^{a}$	$2.54\times10^{-2}\pm1.42\times10^{-2}{}^{a}$	$1.39\times10^{-2}\pm7.09\times10^{-3}{}^{a}$	$2.07\times10^{-2}\pm7.32\times10^{-3}{}^{a}$
Ethyl DL-Leucate	10348-47-7	$C_8H_{16}O_3$	30.16	$3.15\times10^{-3}\pm3.03\times10^{-3}{}^{a}$	$1.80\times10^{-3}\pm9.26\times10^{-4}{}^{a}$	$2.49\times10^{-3}\pm1.46\times10^{-3}{}^{a}$	$4.85\times10^{-3}\pm8.16\times10^{-4}{}^{a}$	$2.36\times10^{-3}\pm1.03\times10^{-3}{}^{a}$
Ethyl caprate	110-38-3	$C_{12}H_{24}O_2$	34.53	$1.82\times10^{-3}\pm7.06\times10^{-4}{}^{b}$	$2.24\times10^{-3}\pm8.53\times10^{-4}{}^{b}$	$2.80\times10^{-3}\pm5.07\times10^{-4}{}^{b}$	$4.24\times10^{-3}\pm1.01\times10^{-3}{}^{a}$	$3.13\times10^{-3}\pm7.77\times10^{-4}{}^{ab}$
Ethyl benzoate	93-89-0	$C_9H_{10}O_2$	35.81	$3.25\times10^{-3}\pm9.84\times10^{-4}{}^{b}$	$3.32\times10^{-3}\pm1.22\times10^{-3}{}^{b}$	$4.25\times10^{-3}\pm1.55\times10^{-3}{}^{b}$	$7.12\times10^{-3}\pm1.81\times10^{-3}{}^{a}$	$6.01\times10^{-3}\pm2.44\times10^{-3}{}^{a}$
Ethyl undecanoate	627-90-7	$C_{13}H_{26}O_2$	36.97	$8.53\times10^{-4}\pm2.97\times10^{-4}{}^{a}$	$2.15\times10^{-3}\pm1.63\times10^{-3}{}^{a}$	$1.72\times10^{-3}\pm1.08\times10^{-3}{}^{a}$	$1.27\times10^{-3}\pm4.17\times10^{-4}{}^{a}$	$2.18\times10^{-3}\pm1.10\times10^{-3}{}^{a}$
Benzyl acetate	140-11-4	$C_9H_{10}O_2$	38.63	ND	$5.73\times10^{-4}\pm1.07\times10^{-4}{}^{a}$	$5.37\times10^{-4}\pm9.82\times10^{-5}{}^{a}$	ND	ND
Methyl salicylate	119-36-8	$C_8H_8O_3$	40.59	$8.92\times10^{-3}\pm3.35\times10^{-3}{}^{ab}$	$6.83\times10^{-3}\pm2.21\times10^{-3}{}^{b}$	$7.27\times10^{-3}\pm1.94\times10^{-3}{}^{ab}$	$1.14\times10^{-2}\pm1.10\times10^{-3}{}^{a}$	$8.17\times10^{-3}\pm2.71\times10^{-3}{}^{ab}$
6-Nonenoic acid, 8-methyl-, 3-methylbutyl ester	1215128-16-7	$C_{15}H_{28}O_2$	42.90	$7.16\times10^{-3}\pm4.26\times10^{-3}{}^{a}$	$9.92\times10^{-3}\pm2.53\times10^{-3}{}^{a}$	$1.16\times10^{-2}\pm3.92\times10^{-3}{}^{a}$	$5.59\times10^{-3}\pm2.06\times10^{-3}{}^{a}$	$9.36\times10^{-3}\pm4.62\times10^{-3}{}^{a}$
Nonanoic acid, 8-methyl-, 4-methylpentyl ester	1215127-97-1	$C_{16}H_{32}O_2$	46.43	$1.48\times10^{-3}\pm1.19\times10^{-3}{}^{a}$	$3.00\times10^{-3}\pm9.36\times10^{-4}{}^{a}$	$2.99\times10^{-3}\pm1.23\times10^{-3}{}^{a}$	$1.61\times10^{-3}\pm2.38\times10^{-4}{}^{a}$	$3.00\times10^{-3}\pm1.61\times10^{-3}{}^{a}$
6-Nonenoic acid, 8-methyl-, 4-methylpentyl ester	1215128-18-9	$C_{16}H_{30}O_2$	47.15	$4.69\times10^{-3}\pm1.89\times10^{-3}{}^{ab}$	$5.10\times10^{-3}\pm1.29\times10^{-3}{}^{ab}$	$6.55\times10^{-3}\pm2.38\times10^{-3}{}^{a}$	$2.38\times10^{-3}\pm5.58\times10^{-4}{}^{b}$	$5.16\times10^{-3}\pm2.51\times10^{-3}{}^{ab}$

Ethyl tetradecanoate	124-06-1	$C_{16}H_{32}O_2$	49.52	$3.09\times10^{-3}\pm1.33\times10^{-3ab}$	$2.54\times10^{-3}\pm1.36\times10^{-3ab}$	$2.32\times10^{-3}\pm1.68\times10^{-3b}$	$5.58\times10^{-3}\pm4.72\times10^{-4a}$	$3.40\times10^{-3}\pm1.88\times10^{-3ab}$
tridecanolide	1725-04-8	$C_{13}H_{24}O_2$	49.62	$1.95\times10^{-3}\pm6.95\times10^{-4a}$	$1.47\times10^{-3}\pm8.57\times10^{-4a}$	$1.60\times10^{-3}\pm6.68\times10^{-4a}$	$1.14\times10^{-3}\pm1.92\times10^{-4a}$	$1.96\times10^{-3}\pm1.22\times10^{-3a}$
9-pentadecenoic acid, ethyl ester	56219-09-1	$C_{17}H_{32}O_2$	50.76	$2.21\times10^{-4}\pm1.21\times10^{-4a}$	ND	$2.32\times10^{-4}\pm1.19\times10^{-4a}$	ND	$1.50\times10^{-4}\pm3.44\times10^{-5ab}$
Pentadecyl Acetate	629-58-3	$C_{17}H_{34}O_2$	51.04	$1.32\times10^{-3}\pm4.18\times10^{-4ab}$	ND	$3.26\times10^{-3}\pm2.32\times10^{-3a}$	ND	$2.47\times10^{-3}\pm1.79\times10^{-3ab}$
Methyl palmitate	112-39-0	$C_{17}H_{34}O_2$	52.03	$5.32\times10^{-4}\pm4.42\times10^{-4b}$	$1.29\times10^{-3}\pm8.97\times10^{-4b}$	$1.77\times10^{-3}\pm1.11\times10^{-3ab}$	$3.11\times10^{-3}\pm1.22\times10^{-3a}$	$2.30\times10^{-3}\pm9.19\times10^{-4ab}$
Ethyl palmitate	628-97-7	$C_{18}H_{36}O_2$	52.51	$7.85\times10^{-3}\pm2.55\times10^{-3b}$	$4.41\times10^{-3}\pm1.44\times10^{-3c}$	$9.13\times10^{-3}\pm2.36\times10^{-3b}$	$1.64\times10^{-2}\pm2.45\times10^{-3a}$	$1.44\times10^{-2}\pm4.39\times10^{-3a}$
9-Hexadecenoic acid,ethyl ester	54546-22-4	$C_{18}H_{34}O_2$	52.85	$7.43\times10^{-4}\pm2.56\times10^{-4ab}$	ND	ND	$1.25\times10^{-3}\pm3.90\times10^{-4a}$	ND
elaidic acid ethyl ester	6114-18-7	$C_{20}H_{38}O_2$	55.11	$7.34\times10^{-4}\pm3.07\times10^{-4b}$	ND	$9.19\times10^{-4}\pm5.49\times10^{-4b}$	$1.99\times10^{-3}\pm8.98\times10^{-4a}$	$1.73\times10^{-3}\pm7.35\times10^{-4ab}$
ethyl linoleate	7619-08-1	$C_{20}H_{36}O_2$	55.63	$2.39\times10^{-3}\pm5.91\times10^{-4b}$	ND	ND	$5.22\times10^{-3}\pm4.41\times10^{-4a}$	$4.26\times10^{-3}\pm1.20\times10^{-3a}$
Ethyl linolenate	1191-41-9	$C_{20}H_{34}O_2$	56.33	$1.78\times10^{-3}\pm5.91\times10^{-4b}$	ND	ND	$3.03\times10^{-3}\pm3.15\times10^{-4a}$	$3.78\times10^{-3}\pm7.38\times10^{-4a}$
Methyl acetate	79-20-9	$C_3H_6O_2$	4.55	$5.65\times10^{-4}\pm1.28\times10^{-4c}$	$7.78\times10^{-4}\pm3.48\times10^{-4bc}$	ND	$9.51\times10^{-4}\pm1.78\times10^{-4b}$	$2.04\times10^{-3}\pm8.43\times10^{-4a}$
Hexyl valerate	1117-59-5	$C_{11}H_{22}O_2$	26.38	ND	ND	ND	$2.13\times10^{-3}\pm1.30\times10^{-3a}$	$2.56\times10^{-3}\pm7.40\times10^{-4a}$
Ethyl salicylate	118-61-6	$C_9H_{10}O_3$	41.98	$4.96\times10^{-3}\pm2.69\times10^{-3b}$	$6.47\times10^{-3}\pm2.04\times10^{-3b}$	$5.97\times10^{-3}\pm2.50\times10^{-3b}$	$1.62\times10^{-2}\pm4.34\times10^{-3a}$	$8.06\times10^{-3}\pm1.65\times10^{-3b}$
Ethyl laurate	106-33-2	$C_{14}H_{28}O_2$	43.34	$2.11\times10^{-3}\pm1.42\times10^{-3a}$	$1.73\times10^{-3}\pm1.12\times10^{-3a}$	$1.76\times10^{-3}\pm6.88\times10^{-4a}$	$3.58\times10^{-3}\pm5.39\times10^{-4a}$	$2.41\times10^{-3}\pm6.73\times10^{-4a}$
Ethyl pentadecanoate	41114-00-5	$C_{17}H_{34}O_2$	50.38	ND	ND	$1.56\times10^{-3}\pm2.24\times10^{-3a}$	ND	ND
2-methylbutyl 2-methylbutanoate	2445-78-5	$C_{10}H_{20}O_2$	17.34	ND	ND	ND	$1.82\times10^{-3}\pm3.39\times10^{-4a}$	ND
Methyl myristate	124-10-7	$C_{15}H_{30}O_2$	48.89	ND	ND	ND	$9.45\times10^{-4}\pm1.11\times10^{-4a}$	$6.96\times10^{-4}\pm1.95\times10^{-4b}$
Propyl acetate	109-60-4	$C_5H_{10}O_2$	6.87	ND	ND	$4.18\times10^{-3}\pm2.63\times10^{-3a}$	ND	ND
propyl hexanoate	626-77-7	$C_9H_{18}O_2$	19.20	ND	ND	$2.37\times10^{-3}\pm1.21\times10^{-3a}$	ND	ND
Isoamyl acetate	123-92-2	$C_7H_{14}O_2$	11.01	ND	ND	ND	$6.02\times10^{-3}\pm2.33\times10^{-3a}$	ND
**Ketone**								
Damascenone	23696-85-7	$C_{13}H_{18}O$	42.29	$2.45\times10^{-3}\pm1.16\times10^{-3b}$	$3.21\times10^{-3}\pm1.42\times10^{-3ab}$	$3.51\times10^{-3}\pm8.13\times10^{-4ab}$	ND	$4.25\times10^{-3}\pm9.34\times10^{-4a}$
Dihydro-beta-ionone	17283-81-7	$C_{13}H_{22}O$	42.70	$5.28\times10^{-4}\pm1.51\times10^{-4c}$	$1.11\times10^{-3}\pm4.85\times10^{-4b}$	ND	$1.33\times10^{-3}\pm1.77\times10^{-4ab}$	$1.52\times10^{-3}\pm1.98\times10^{-4a}$

β-Ionone	14901-07-6	$C_{13}H_{20}O$	47.05	$1.50\times10^{-3}\pm9.08\times10^{-4b}$	ND	ND	$5.36\times10^{-3}\pm1.79\times10^{-3a}$	ND
**Acid**								
Acetic acid	64-19-7	$C_2H_4O_2$	25.77	$2.07\times10^{-2}\pm5.05\times10^{-3b}$	$1.85\times10^{-2}\pm9.15\times10^{-3b}$	$2.21\times10^{-2}\pm1.11\times10^{-2b}$	$2.85\times10^{-2}\pm5.79\times10^{-3ab}$	$3.54\times10^{-2}\pm3.89\times10^{-3a}$
(6E)-8-Methyl-6-nonenoic acid	59320-77-3	$C_{10}H_{18}O_2$	33.20	$3.17\times10^{-2}\pm1.42\times10^{-2ab}$	$4.66\times10^{-2}\pm3.14\times10^{-2a}$	$3.42\times10^{-2}\pm1.74\times10^{-2ab}$	$1.67\times10^{-2}\pm1.99\times10^{-2b}$	$5.44\times10^{-2}\pm7.21\times10^{-3a}$
4-Methylpentanoic acid	646-07-1	$C_6H_{12}O_2$	41.76	$3.58\times10^{-3}\pm1.54\times10^{-3a}$	$2.88\times10^{-3}\pm1.34\times10^{-3ab}$	ND	$4.22\times10^{-3}\pm9.60\times10^{-4a}$	ND
Hexanoic acid	142-62-1	$C_6H_{12}O_2$	43.49	$6.50\times10^{-2}\pm2.10\times10^{-2a}$	$6.44\times10^{-2}\pm2.28\times10^{-2a}$	$6.09\times10^{-2}\pm3.23\times10^{-2a}$	$6.35\times10^{-2}\pm1.25\times10^{-2a}$	$5.98\times10^{-2}\pm1.25\times10^{-2a}$
Heptanoic acid	111-14-8	$C_7H_{14}O_2$	47.70	$9.40\times10^{-4}\pm1.67\times10^{-4a}$	$9.32\times10^{-4}\pm3.30\times10^{-4a}$	$9.30\times10^{-4}\pm3.93\times10^{-4a}$	$1.01\times10^{-3}\pm6.24\times10^{-5a}$	$8.33\times10^{-4}\pm3.22\times10^{-4a}$
Octanoic acid	124-07-2	$C_8H_{16}O_2$	49.74	$2.98\times10^{-3}\pm1.25\times10^{-3a}$	$3.58\times10^{-3}\pm1.99\times10^{-3a}$	$2.64\times10^{-3}\pm5.74\times10^{-4a}$	$3.84\times10^{-3}\pm5.43\times10^{-4a}$	$2.31\times10^{-3}\pm3.39\times10^{-4a}$
7-Methyloctanoic acid	693-19-6	$C_9H_{18}O_2$	50.65	$2.22\times10^{-3}\pm4.54\times10^{-4a}$	$2.22\times10^{-3}\pm4.04\times10^{-4a}$	$2.00\times10^{-3}\pm4.97\times10^{-4a}$	$2.87\times10^{-3}\pm3.39\times10^{-4a}$	$2.45\times10^{-3}\pm6.07\times10^{-4a}$
Nonanoic acid	112-05-0	$C_9H_{18}O_2$	51.37	$1.65\times10^{-3}\pm5.07\times10^{-4a}$	$8.64\times10^{-4}\pm3.79\times10^{-4b}$	$1.27\times10^{-3}\pm2.75\times10^{-4ab}$	$1.75\times10^{-3}\pm1.71\times10^{-4a}$	$1.05\times10^{-3}\pm3.79\times10^{-4b}$
8-methylnonanoic acid	5963-14-4	$C_{10}H_{20}O_2$	52.14	$2.45\times10^{-3}\pm5.52\times10^{-4a}$	$2.37\times10^{-3}\pm5.77\times10^{-4a}$	$3.19\times10^{-3}\pm7.36\times10^{-4a}$	$3.04\times10^{-3}\pm8.38\times10^{-4a}$	$3.43\times10^{-3}\pm1.19\times10^{-3a}$
Benzoic acid	65-85-0	$C_7H_6O_2$	54.78	$5.41\times10^{-4}\pm9.14\times10^{-5b}$	ND	$1.23\times10^{-3}\pm4.02\times10^{-4a}$	ND	ND
Lauric acid	143-07-7	$C_{12}H_{24}O_2$	55.16	$7.70\times10^{-4}\pm2.13\times10^{-4a}$	$5.07\times10^{-4}\pm1.89\times10^{-4ab}$	$5.71\times10^{-4}\pm8.57\times10^{-5ab}$	ND	ND
(Z)-8-methylnon-6-enoic acid	21382-25-2	$C_{10}H_{18}O_2$	52.38	$6.11\times10^{-3}\pm2.36\times10^{-3a}$	$7.14\times10^{-3}\pm2.14\times10^{-3a}$	ND	ND	$8.07\times10^{-3}\pm1.78\times10^{-3a}$
Decanoic acid	334-48-5	$C_{10}H_{20}O_2$	52.77	$7.23\times10^{-4}\pm2.26\times10^{-4a}$	$6.22\times10^{-4}\pm1.28\times10^{-4ab}$	ND	ND	ND
Myristic acid	544-63-8	$C_{14}H_{28}O_2$	57.26	$5.40\times10^{-4}\pm8.24\times10^{-5a}$	ND	ND	ND	$5.73\times10^{-4}\pm1.06\times10^{-4a}$
2-Methylbutyric acid	116-53-0	$C_5H_{10}O_2$	36.00	ND	ND	ND	$8.67\times10^{-3}\pm8.08\times10^{-4a}$	$9.05\times10^{-3}\pm1.90\times10^{-3a}$
2,4-Dimethylpentanoic acid	5868-33-7	$C_7H_{14}O_2$	36.01	ND	ND	$2.43\times10^{-3}\pm1.78\times10^{-3a}$	ND	$7.15\times10^{-4}\pm1.08\times10^{-4b}$
Propanoic acid	79-09-4	$C_3H_6O_2$	30.02	$2.07\times10^{-2}\pm5.05\times10^{-3a}$	$1.85\times10^{-2}\pm9.15\times10^{-3a}$	$2.21\times10^{-2}\pm1.11\times10^{-2a}$	$2.85\times10^{-2}\pm5.79\times10^{-3a}$	$3.54\times10^{-2}\pm3.89\times10^{-3a}$
**Aldehyde**								
Hexanal	66-25-1	$C_6H_{12}O$	9.86	$1.71\times10^{-3}\pm6.95\times10^{-4a}$	$1.32\times10^{-3}\pm7.64\times10^{-4ab}$	$8.23\times10^{-4}\pm2.16\times10^{-4b}$	ND	$1.92\times10^{-3}\pm1.06\times10^{-3a}$

Octanal	124-13-0	$C_8H_{16}O$	17.91	$1.11\times10^{-2}\pm6.06\times10^{-3}$[a]	$8.14\times10^{-3}\pm6.13\times10^{-3}$[a]	$7.96\times10^{-3}\pm5.12\times10^{-3}$[a]	$9.37\times10^{-3}\pm5.58\times10^{-3}$[a]	$5.75\times10^{-3}\pm1.79\times10^{-3}$[a]
Nonanal	124-19-6	$C_9H_{18}O$	22.97	$2.92\times10^{-2}\pm1.74\times10^{-2}$[a]	$2.09\times10^{-2}\pm1.69\times10^{-2}$[a]	$1.96\times10^{-2}\pm1.19\times10^{-2}$[a]	$3.11\times10^{-2}\pm6.73\times10^{-3}$[a]	$1.65\times10^{-2}\pm6.78\times10^{-3}$[a]
Benzaldehyde	100-52-7	C_7H_6O	29.33	$2.09\times10^{-3}\pm4.87\times10^{-4}$[a]	$1.80\times10^{-3}\pm8.80\times10^{-4}$[a]	$1.95\times10^{-3}\pm1.26\times10^{-3}$[a]	$3.24\times10^{-3}\pm1.77\times10^{-3}$[a]	$2.26\times10^{-3}\pm1.63\times10^{-3}$[a]
Phenylacetaldehyde	122-78-1	C_8H_8O	35.00	$3.18\times10^{-3}\pm1.55\times10^{-3}$[a]	$5.58\times10^{-3}\pm2.54\times10^{-3}$[a]	$4.27\times10^{-3}\pm1.50\times10^{-3}$[a]	ND	$4.33\times10^{-3}\pm2.19\times10^{-3}$[a]
13-Methyltetradecanal	75853-51-9	$C_{15}H_{30}O$	48.26	$2.97\times10^{-3}\pm1.15\times10^{-3}$[a]	$3.34\times10^{-3}\pm7.13\times10^{-4}$[a]	$5.00\times10^{-3}\pm2.62\times10^{-3}$[a]	$4.62\times10^{-3}\pm1.62\times10^{-3}$[a]	$4.76\times10^{-3}\pm2.11\times10^{-3}$[a]
(2E)-2-Decenal	3913-81-3	$C_{10}H_{18}O$	34.77	ND	$1.60\times10^{-3}\pm1.44\times10^{-3}$[a]	ND	ND	$1.16\times10^{-3}\pm3.64\times10^{-4}$[a]
**Alkane**								
Dodecane, 3-methyl-	17312-57-1	$C_{13}H_{28}$	21.13	$5.65\times10^{-2}\pm2.42\times10^{-2}$[a]	$4.72\times10^{-2}\pm2.36\times10^{-2}$[a]	ND	ND	ND
2-methyltetradecane	1560-95-8	$C_{15}H_{32}$	26.05	$4.66\times10^{-2}\pm2.26\times10^{-2}$[a]	$3.48\times10^{-2}\pm1.69\times10^{-2}$[a]	$7.04\times10^{-2}\pm3.14\times10^{-2}$[a]	$3.90\times10^{-2}\pm1.51\times10^{-2}$[a]	$5.48\times10^{-2}\pm3.10\times10^{-2}$[a]
Pentadecane	629-62-9	$C_{15}H_{32}$	28.12	$1.46\times10^{-2}\pm1.01\times10^{-2}$[a]	$1.39\times10^{-2}\pm7.83\times10^{-3}$[a]	$1.36\times10^{-2}\pm3.78\times10^{-3}$[a]	$1.15\times10^{-2}\pm2.25\times10^{-3}$[a]	$1.56\times10^{-2}\pm4.99\times10^{-3}$[a]
Pentadecane, 2-methyl-	1560-93-6	$C_{16}H_{34}$	30.84	$1.65\times10^{-2}\pm7.61\times10^{-3}$[a]	$1.41\times10^{-2}\pm6.51\times10^{-3}$[a]	$1.72\times10^{-2}\pm8.59\times10^{-3}$[a]	$1.96\times10^{-2}\pm2.48\times10^{-3}$[a]	$1.62\times10^{-2}\pm5.24\times10^{-3}$[a]
Pentadecane, 3-methyl-	2882-96-4	$C_{16}H_{34}$	31.37	$1.14\times10^{-3}\pm8.15\times10^{-4}$[b]	$3.64\times10^{-3}\pm2.38\times10^{-3}$[a]	$2.49\times10^{-3}\pm3.32\times10^{-3}$[ab]	ND	$4.49\times10^{-4}\pm2.46\times10^{-4}$[b]
Hexadecane	544-76-3	$C_{16}H_{34}$	32.84	$8.70\times10^{-3}\pm6.48\times10^{-3}$[a]	$1.00\times10^{-2}\pm6.58\times10^{-3}$[a]	$9.31\times10^{-3}\pm5.20\times10^{-3}$[a]	$7.11\times10^{-3}\pm1.97\times10^{-3}$[a]	$1.40\times10^{-2}\pm4.88\times10^{-3}$[a]
2-methylhexadecane	1560-92-5	$C_{17}H_{36}$	35.42	$1.00\times10^{-2}\pm4.99\times10^{-3}$[a]	$7.51\times10^{-3}\pm4.38\times10^{-3}$[a]	ND	ND	$8.78\times10^{-3}\pm3.59\times10^{-3}$[a]
Heptadecane	629-78-7	$C_{17}H_{36}$	37.33	$1.07\times10^{-2}\pm8.25\times10^{-3}$[a]	$6.01\times10^{-3}\pm4.14\times10^{-3}$[a]	$7.72\times10^{-3}\pm5.34\times10^{-3}$[a]	ND	$8.84\times10^{-3}\pm3.97\times10^{-3}$[a]
2-methyltridecane	1560-96-9	$C_{14}H_{30}$	21.11	$2.45\times10^{-2}\pm1.29\times10^{-2}$[b]	$6.65\times10^{-2}\pm3.50\times10^{-2}$[a]	$4.62\times10^{-2}\pm1.46\times10^{-2}$[ab]	ND	$7.21\times10^{-2}\pm3.57\times10^{-2}$[a]
**Terpene**								
Styrene	100-42-5	C_8H_8	16.47	$4.34\times10^{-3}\pm7.95\times10^{-4}$[ab]	$3.33\times10^{-3}\pm1.22\times10^{-3}$[b]	$3.38\times10^{-3}\pm2.35\times10^{-3}$[ab]	$5.79\times10^{-3}\pm1.48\times10^{-3}$[a]	$4.48\times10^{-3}\pm1.74\times10^{-3}$[ab]
(E)-4,8-Dimethylnona-1,3,7-triene	19945-61-0	$C_{11}H_{18}$	18.55	$6.31\times10^{-3}\pm2.66\times10^{-3}$[a]	$5.26\times10^{-3}\pm5.47\times10^{-3}$[a]	$4.63\times10^{-3}\pm3.13\times10^{-3}$[a]	$4.11\times10^{-3}\pm8.29\times10^{-4}$[a]	$5.77\times10^{-3}\pm1.84\times10^{-3}$[a]
(E)-tetradec-6-ene	41446-64-4	$C_{14}H_{28}$	22.09	ND	$2.58\times10^{-3}\pm1.01\times10^{-3}$[a]	$2.57\times10^{-3}\pm1.08\times10^{-3}$[a]	$2.00\times10^{-3}\pm5.88\times10^{-4}$[a]	ND
2-methyl-1-tetradecene	52254-38-3	$C_{15}H_{30}$	26.67	$5.25\times10^{-2}\pm3.27\times10^{-2}$[a]	$2.49\times10^{-2}\pm1.47\times10^{-2}$[a]	$4.98\times10^{-2}\pm2.30\times10^{-2}$[a]	$4.34\times10^{-2}\pm1.37\times10^{-2}$[a]	$3.53\times10^{-2}\pm1.71\times10^{-2}$[a]
(+)-longicyclene	1137-12-8	$C_{15}H_{24}$	27.40	ND	$9.96\times10^{-4}\pm3.11\times10^{-4}$[a]	$1.55\times10^{-3}\pm6.16\times10^{-4}$[a]	ND	$1.45\times10^{-3}\pm2.30\times10^{-4}$[a]

γ-himachalene	53111-25-4	$C_{15}H_{24}$	36.48	$4.67\times10^{-2}\pm3.44\times10^{-2}{}^{a}$	$4.69\times10^{-2}\pm3.89\times10^{-2}{}^{a}$	$2.76\times10^{-2}\pm2.07\times10^{-2}{}^{a}$	$5.67\times10^{-2}\pm4.15\times10^{-2}{}^{a}$	$2.07\times10^{-2}\pm1.49\times10^{-2}{}^{a}$
beta-Longipinene	41432-70-6	$C_{15}H_{24}$	37.75	ND	ND	$8.09\times10^{-3}\pm2.38\times10^{-3}{}^{a}$	$7.91\times10^{-3}\pm3.15\times10^{-3}{}^{a}$	$6.78\times10^{-3}\pm3.01\times10^{-3}{}^{a}$
2-epi-trans-β-caryophyllene	68832-35-9	$C_{15}H_{24}$	37.76	ND	$6.67\times10^{-3}\pm4.53\times10^{-3}{}^{a}$	ND	ND	ND
(3E,7E)-4,8,12-Trimethyl-1,3,7,11-tridecatetraene	62235-06-7	$C_{16}H_{26}$	41.82	ND	ND	$5.00\times10^{-3}\pm7.22\times10^{-4}{}^{a}$	ND	$5.77\times10^{-3}\pm1.08\times10^{-3}{}^{a}$
(+)-Limonene	5989-27-5	$C_{10}H_{16}$	13.93	ND	$9.72\times10^{-3}\pm3.55\times10^{-3}{}^{a}$	$5.21\times10^{-3}\pm2.59\times10^{-3}{}^{a}$	ND	ND
8-Heptadecene	2579-04-6	$C_{17}H_{34}$	39.31	ND	ND	ND	ND	$1.00\times10^{-3}\pm7.00\times10^{-4}{}^{a}$
zingiberene	495-60-3	$C_{15}H_{24}$	37.95	ND	ND	$1.35\times10^{-3}\pm1.16\times10^{-3}{}^{a}$	ND	ND
**Others**								
Ethylbenzene	100-41-4	C_8H_{10}	11.20	$1.54\times10^{-3}\pm4.17\times10^{-4}{}^{a}$	ND	ND	ND	$2.26\times10^{-3}\pm1.13\times10^{-3}{}^{a}$
p-Xylene	106-42-3	C_8H_{10}	11.77	$2.32\times10^{-3}\pm1.21\times10^{-3}{}^{a}$	$1.83\times10^{-3}\pm5.74\times10^{-4}{}^{ab}$	$1.01\times10^{-3}\pm3.38\times10^{-4}{}^{b}$	ND	ND
m-Xylene	108-38-3	C_8H_{10}	13.50	$5.38\times10^{-4}\pm1.32\times10^{-4}{}^{a}$	$5.90\times10^{-4}\pm2.33\times10^{-4}{}^{a}$	$9.05\times10^{-4}\pm3.51\times10^{-4}{}^{a}$	ND	$7.14\times10^{-4}\pm1.41\times10^{-4}{}^{a}$
2-Methoxy-3-isobutyl pyrazine	24683-00-9	$C_9H_{14}N_2O$	29.20	$2.46\times10^{-3}\pm8.64\times10^{-4}{}^{a}$	$2.75\times10^{-3}\pm5.03\times10^{-4}{}^{a}$	$2.01\times10^{-3}\pm9.99\times10^{-4}{}^{ab}$	$1.72\times10^{-3}\pm7.52\times10^{-4}{}^{ab}$	$1.10\times10^{-3}\pm1.13\times10^{-3}{}^{b}$
Guaiacol	90-05-1	$C_7H_8O_2$	44.22	$2.74\times10^{-3}\pm1.16\times10^{-3}{}^{a}$	$3.07\times10^{-3}\pm2.19\times10^{-3}{}^{a}$	$1.66\times10^{-3}\pm4.65\times10^{-4}{}^{a}$	$2.80\times10^{-3}\pm4.43\times10^{-4}{}^{a}$	$2.01\times10^{-3}\pm4.09\times10^{-4}{}^{a}$
4-Ethyl-2-methoxyphenol	2785-89-9	$C_9H_{12}O_2$	49.33	$1.56\times10^{-3}\pm4.98\times10^{-4}{}^{b}$	$6.07\times10^{-3}\pm2.71\times10^{-3}{}^{b}$	$3.22\times10^{-3}\pm9.82\times10^{-4}{}^{a}$	$3.54\times10^{-3}\pm1.69\times10^{-3}{}^{b}$	$2.58\times10^{-2}\pm4.28\times10^{-3}{}^{a}$
4-Ethylphenol	123-07-9	$C_8H_{10}O$	51.59	ND	$2.62\times10^{-3}\pm3.07\times10^{-3}{}^{ab}$	ND	$1.46\times10^{-3}\pm5.12\times10^{-4}{}^{b}$	$4.43\times10^{-3}\pm7.73\times10^{-4}{}^{a}$

Note: Different superscript letters within the same row indicate significant differences among groups ($p < 0.05$, Kruskal-Wallis test followed by Dunn's post-hoc test). Compounds without superscript letters showed no significant differences among groups ($p \geq 0.05$). Data are shown as mean ± SD (n=5). RT: Retention index calculated for TG-WAXMS B capillary column (30 m × 0.25 mm × 0.25 µm). ND: not detected.

Supplementary Table S2. Aroma characteristics and ROAV values of key volatile compounds in different treatment groups

Name of volatile compounds	CAS	Formula	Aroma descriptor	Threshold (μg/L)	ROAV (%)				
					Y0	Y5	Y15	Y25	Y50
**Alcohol**									
Linalool	78-70-6	$C_{10}H_{18}O$	floral, green	6	0.1701	0.1855	0.2023	0.3721	0.2463
(2R,3R)-(-)-2,3-Butanediol	24347-58-8	$C_4H_{10}O_2$	faint sweet, alcoholic note，slight creamy	0.002	100.0000	-	-	-	-
Ester									
tridecanolide	1725-04-8	$C_{13}H_{24}O_2$	milky, creamy，sweet, buttery nuance	0.5	0.1147	0.1069	0.1592	0.1326	0.1845
ethyl 4-methylpentanoate	25415-67-2	$C_8H_{16}O_2$	fruity	1	0.1321	-	0.1667	0.3808	0.2442
ethyl isovalerate	108-64-5	$C_7H_{14}O_2$	strawberry, candy, fruity	0.1	0.2382	0.2825	0.6318	1.1512	0.7671
methyl hexanoate	106-70-7	$C_7H_{14}O_2$	ethereal, fruity, pineapple, apricot, strawberry, tropical fruit, banana, bacon	0.3	-	0.1576	-	0.2267	0.2573
Ethyl Acetate	141-78-6	$C_4H_8O_2$	ethereal, fruity, sweet, weedy, green	10	-	-	0.1706	0.2791	0.2320
ethyl nonanoate	123-29-5	$C_{11}H_{22}O_2$	fruity, rose, waxy, rum, wine, natural, tropical	10	-	-	-	0.1221	-
Ethyl caprylate	106-32-1	$C_{10}H_{20}O_2$	fruity, wine, waxy, sweet, apricot, banana, brandy, pear	5	-	-	-	0.1709	-
Isoamyl acetate	123-92-2	$C_7H_{14}O_2$	sweet, fruity, banana, solvent	2	-	-	-	0.1750	-
2-methylbutyl 2-methylbutanoate	2445-78-5	$C_{10}H_{20}O_2$	sweet, fruity, ester, berry, green, waxy, apple	0.3	-	-	-	0.3527	-
**Ketone**									
β-Ionone	14901-07-6	$C_{13}H_{20}O$	floral, woody, sweet, fruity, berry, tropical, beeswax	0.007	6.3025	-	-	44.5183	-
Damascenone	23696-85-7	$C_{13}H_{18}O$	natural, sweet, fruity, rose, plum, grape, raspberry, sugar	0.002	36.0294	58.3636	87.3134	-	100.0000
Dihydro-beta-ionone	17283-81-7	$C_{13}H_{22}O$	earthy, woody, mahogany, orris, dry amber	0.3	-	0.1345	-	0.2578	0.2384
**Acid**									
Hexanoic acid	142-62-1	$C_6H_{12}O_2$	cheesy, fruity, phenolic, fatty, goaty	10	0.1912	0.2342	0.3030	0.3692	0.2814
**Aldehyde**									

Nonanal	124-19-6	$C_9H_{18}O$	waxy, aldehydic, rose, fresh orris, orange peel, fatty peely	3	0.2863	0.2533	0.3250	0.6027	0.2588
Octanal	124-13-0	$C_8H_{16}O$	aldehydic, waxy, citrus, orange peel, green herbal, fresh, fatty	0.587	0.5562	0.5043	0.6747	0.9281	0.4610
(2E)-2-Decenal	3913-81-3	$C_{10}H_{18}O$	waxy, fatty, earthy, green, cilantro, mushroom, aldehydic, fried chicken, fat tallow	0.3	-	0.1939	-	-	0.1820
**Heterocyclic compounds**									
2-Methoxy-3-isobutyl pyrazine	24683-00-9	$C_9H_{14}N_2O$	green pea, green bell pepper, green pea galbanum	0.001	72.3529	100.0000	100.0000	100.0000	51.7647
**Phenolic compounds**									
4-Ethyl-2-methoxyphenol	2785-89-9	C_9H_{12}O2	spicy, smoky, bacon, phenolic, clove	5	-	-	-	-	0.2428

Note: - = Not detected/Not calculated; ROAV = Relative Odor Activity Value

Supplementary Table S3. ^{1}H-NMR-derived concentrations (mmol/L, mean ± SD) of non-volatile metabolites in pickled chili peppers fermented with brine aged for 0, 5, 15, 25, and 50 years (n=5).

Compound Name	Chemical Shift (ppm)	Multiplicity	Groups				
			Y0	Y5	Y15	Y25	Y50
**Amino Acids and Derivatives**							
Tryptophan	7.7246	m	$8.45\times10^{-2}\pm3.28\times10^{-2\mathrm{a}}$	$7.27\times10^{-2}\pm1.11\times10^{-2\mathrm{a}}$	$7.45\times10^{-2}\pm8.78\times10^{-3\mathrm{a}}$	$8.16\times10^{-2}\pm2.27\times10^{-2\mathrm{a}}$	$8.73\times10^{-2}\pm8.39\times10^{-3\mathrm{a}}$
Phenylalanine	7.3740	m	$5.59\times10^{-1}\pm5.54\times10^{-2\mathrm{a}}$	$4.83\times10^{-1}\pm2.81\times10^{-2\mathrm{a}}$	$5.23\times10^{-1}\pm3.05\times10^{-2\mathrm{a}}$	$5.37\times10^{-1}\pm5.61\times10^{-2\mathrm{a}}$	$5.41\times10^{-1}\pm2.06\times10^{-2\mathrm{a}}$
Tyramine	7.2144	d	$1.34\times10^{-1}\pm4.91\times10^{-2\mathrm{b}}$	$1.63\times10^{-1}\pm2.12\times10^{-2\mathrm{b}}$	$3.71\times10^{-1}\pm3.40\times10^{-2\mathrm{a}}$	$2.00\times10^{-1}\pm1.21\times10^{-2\mathrm{b}}$	$9.66\times10^{-2}\pm7.23\times10^{-2\mathrm{b}}$
Threonine	4.2506	m	$7.08\times10^{-1}\pm1.87\times10^{-2\mathrm{a}}$	$5.86\times10^{-1}\pm3.49\times10^{-2\mathrm{a}}$	$7.67\times10^{-1}\pm3.27\times10^{-2\mathrm{a}}$	$7.16\times10^{-1}\pm5.61\times10^{-2\mathrm{a}}$	$6.87\times10^{-1}\pm4.48\times10^{-2\mathrm{a}}$
Anserine	7.1342	m	$2.57\times10^{-1}\pm7.99\times10^{-2\mathrm{a}}$	$2.21\times10^{-1}\pm1.61\times10^{-2\mathrm{a}}$	$2.96\times10^{-1}\pm4.34\times10^{-2\mathrm{a}}$	$2.20\times10^{-1}\pm6.84\times10^{-2\mathrm{a}}$	$1.91\times10^{-1}\pm7.57\times10^{-2\mathrm{a}}$
Pyroglutamate	4.1547	m	$8.04\times10^{-1}\pm3.07\times10^{-1\mathrm{a}}$	$1.02\pm1.89\times10^{-1\mathrm{a}}$	$9.31\times10^{-1}\pm2.98\times10^{-1\mathrm{a}}$	$9.44\times10^{-1}\pm1.41\times10^{-1\mathrm{a}}$	$8.77\times10^{-1}\pm1.37\times10^{-1\mathrm{a}}$
Serine	3.9741	m	$1.21\pm2.66\times10^{-1\mathrm{a}}$	$1.33\pm7.03\times10^{-2\mathrm{a}}$	$1.44\pm1.79\times10^{-1\mathrm{a}}$	$8.00\times10^{-1}\pm5.48\times10^{-1\mathrm{a}}$	$1.51\pm7.48\times10^{-2\mathrm{a}}$
Glycine	3.5422	s	$3.56\times10^{-1}\pm2.43\times10^{-1\mathrm{a}}$	$1.92\times10^{-1}\pm9.53\times10^{-2\mathrm{a}}$	$9.55\times10^{-2}\pm1.66\times10^{-2\mathrm{a}}$	$2.21\times10^{-1}\pm1.00\times10^{-1\mathrm{a}}$	$2.31\times10^{-1}\pm5.95\times10^{-2\mathrm{a}}$
Proline	3.3225	m	$5.49\times10^{-1}\pm7.60\times10^{-2\mathrm{a}}$	$5.24\times10^{-1}\pm1.35\times10^{-1\mathrm{a}}$	$5.23\times10^{-1}\pm9.23\times10^{-2\mathrm{a}}$	$5.08\times10^{-1}\pm5.97\times10^{-2\mathrm{a}}$	$4.84\times10^{-1}\pm9.39\times10^{-2\mathrm{a}}$
Ornithine	3.0522	m	$5.06\times10^{-1}\pm1.73\times10^{-2\mathrm{a}}$	$1.00\pm3.95\times10^{-1\mathrm{a}}$	$8.87\times10^{-1}\pm8.51\times10^{-2\mathrm{a}}$	$8.07\times10^{-1}\pm9.61\times10^{-2\mathrm{a}}$	$8.25\times10^{-1}\pm1.13\times10^{-1\mathrm{a}}$
Asparagine	2.9444	m	$3.79\pm2.32\times10^{-1\mathrm{a}}$	$3.63\pm3.00\times10^{-1\mathrm{a}}$	$3.38\pm3.28\times10^{-1\mathrm{a}}$	$3.67\pm1.93\times10^{-1\mathrm{a}}$	$3.35\pm2.25\times10^{-1\mathrm{a}}$
Aspartate	2.7802	m	$1.98\times10^{-1}\pm2.38\times10^{-1\mathrm{ab}}$	$3.20\times10^{-2}\pm6.97\times10^{-3\mathrm{b}}$	$4.66\times10^{-2}\pm1.39\times10^{-2\mathrm{b}}$	$5.51\times10^{-1}\pm3.84\times10^{-2\mathrm{a}}$	$2.74\times10^{-2}\pm3.92\times10^{-3\mathrm{b}}$
4-Aminobutyrate	2.2842	m	$2.05\pm1.80\times10^{-1\mathrm{a}}$	$1.98\pm1.30\times10^{-1\mathrm{a}}$	$1.88\pm9.61\times10^{-2\mathrm{a}}$	$2.26\pm1.61\times10^{-2\mathrm{a}}$	$2.10\pm1.49\times10^{-1\mathrm{a}}$
Methionine	2.1264	m	$2.92\times10^{-1}\pm3.75\times10^{-2\mathrm{a}}$	$2.19\times10^{-1}\pm2.38\times10^{-2\mathrm{a}}$	$3.13\times10^{-1}\pm1.11\times10^{-1\mathrm{a}}$	$2.48\times10^{-1}\pm6.88\times10^{-2\mathrm{a}}$	$2.70\times10^{-1}\pm7.51\times10^{-2\mathrm{a}}$
N-Acetylaspartate	2.0101	s	$1.78\times10^{-1}\pm1.57\times10^{-2\mathrm{a}}$	$2.62\times10^{-1}\pm3.57\times10^{-2\mathrm{a}}$	$1.79\times10^{-1}\pm1.56\times10^{-2\mathrm{a}}$	$1.31\times10^{-1}\pm4.36\times10^{-2\mathrm{a}}$	$1.75\times10^{-1}\pm3.59\times10^{-2\mathrm{a}}$
Alanine	1.4730	d	$1.62\pm4.91\times10^{-1\mathrm{ab}}$	$1.84\pm1.08\times10^{-1\mathrm{ab}}$	$2.16\pm1.81\times10^{-1\mathrm{a}}$	$1.17\pm6.40\times10^{-2\mathrm{b}}$	$2.13\pm1.58\times10^{-1\mathrm{ab}}$
Valine	0.9701	d	$7.20\times10^{-1}\pm2.66\times10^{-2\mathrm{a}}$	$7.20\times10^{-1}\pm3.65\times10^{-2\mathrm{a}}$	$8.17\times10^{-1}\pm3.45\times10^{-2\mathrm{a}}$	$7.23\times10^{-1}\pm6.49\times10^{-2\mathrm{a}}$	$7.47\times10^{-1}\pm4.09\times10^{-2\mathrm{a}}$

Leucine	0.9460	d	$4.99\times10^{-1}\pm3.73\times10^{-2}{}^{a}$	$5.38\times10^{-1}\pm3.72\times10^{-2}{}^{a}$	$6.93\times10^{-1}\pm1.22\times10^{-1}{}^{a}$	$5.65\times10^{-1}\pm5.77\times10^{-2}{}^{a}$	$5.67\times10^{-1}\pm4.63\times10^{-2}{}^{a}$
Isoleucine	0.9246	d	$3.88\times10^{-1}\pm4.21\times10^{-2}{}^{a}$	$4.09\times10^{-1}\pm2.03\times10^{-2}{}^{a}$	$5.26\times10^{-1}\pm1.16\times10^{-1}{}^{a}$	$3.97\times10^{-1}\pm4.90\times10^{-2}{}^{a}$	$4.41\times10^{-1}\pm2.61\times10^{-2}{}^{a}$
N-Isovaleroylglycine	0.9122	t	$8.87\times10^{-2}\pm2.06\times10^{-2}{}^{a}$	$1.01\times10^{-1}\pm1.25\times10^{-2}{}^{a}$	$1.49\times10^{-1}\pm7.09\times10^{-2}{}^{a}$	$8.76\times10^{-2}\pm1.75\times10^{-2}{}^{a}$	$1.02\times10^{-1}\pm1.13\times10^{-2}{}^{a}$
**Organic Acids**							
Formate	8.4439	s	$1.68\times10^{-1}\pm3.05\times10^{-2}{}^{a}$	$1.60\times10^{-1}\pm1.39\times10^{-2}{}^{a}$	$1.22\times10^{-1}\pm1.33\times10^{-2}{}^{b}$	$1.28\times10^{-1}\pm9.86\times10^{-3}{}^{b}$	$1.07\times10^{-1}\pm7.82\times10^{-3}{}^{b}$
Tartrate	4.3096	s	$5.00\times10^{-2}\pm1.32\times10^{-2}{}^{a}$	$5.38\times10^{-2}\pm4.55\times10^{-3}{}^{a}$	$3.97\times10^{-2}\pm3.32\times10^{-3}{}^{a}$	$4.73\times10^{-2}\pm7.17\times10^{-3}{}^{a}$	$5.77\times10^{-2}\pm7.96\times10^{-3}{}^{a}$
Malonate	3.1151	s	$6.10\times10^{-1}\pm1.81\times10^{-1}{}^{a}$	$5.07\times10^{-1}\pm5.26\times10^{-2}{}^{a}$	$2.30\times10^{-1}\pm4.39\times10^{-2}{}^{a}$	$4.87\times10^{-1}\pm1.39\times10^{-1}{}^{a}$	$5.26\times10^{-1}\pm5.07\times10^{-2}{}^{a}$
Succinate	2.3903	s	$1.35\pm3.69\times10^{-1}{}^{b}$	$2.53\pm5.49\times10^{-1}{}^{a}$	$2.21\pm1.50\times10^{-1}{}^{ab}$	$1.53\pm4.85\times10^{-1}{}^{b}$	$2.14\pm2.08\times10^{-1}{}^{ab}$
Pyruvate	2.3642	s	$5.67\times10^{-2}\pm1.80\times10^{-2}{}^{a}$	$3.80\times10^{-2}\pm9.13\times10^{-3}{}^{a}$	$5.39\times10^{-2}\pm9.63\times10^{-3}{}^{a}$	$3.83\times10^{-2}\pm1.15\times10^{-2}{}^{a}$	$3.54\times10^{-2}\pm6.79\times10^{-3}{}^{a}$
Levulinate	2.2245	m	$4.37\times10^{-2}\pm6.69\times10^{-3}{}^{a}$	$4.19\times10^{-2}\pm5.25\times10^{-3}{}^{a}$	$3.57\times10^{-2}\pm5.26\times10^{-3}{}^{a}$	$3.93\times10^{-2}\pm2.29\times10^{-3}{}^{a}$	$3.89\times10^{-2}\pm3.83\times10^{-3}{}^{a}$
Acetate	1.9060	s	23.7 ± 2.45^{ab}	27.9 ± 2.31^{a}	23.0 ± 2.86^{ab}	15.8 ± 5.63^{b}	22.1 ± 2.10^{ab}
Lactate	1.3091	d	33.1 ± 7.42^{ab}	30.7 ± 4.15^{b}	37.6 ± 2.05^{a}	33.4 ± 9.04^{ab}	25.8 ± 1.80^{b}
Butyrate	0.8793	t	$2.76\times10^{-2}\pm4.07\times10^{-3}{}^{b}$	$3.78\times10^{-2}\pm6.71\times10^{-3}{}^{b}$	$1.71\pm6.18\times10^{-1}{}^{a}$	$3.72\times10^{-1}\pm5.91\times10^{-1}{}^{b}$	$6.32\times10^{-2}\pm3.75\times10^{-3}{}^{b}$
2-Hydroxyisovalerate	0.8145	d	$1.92\times10^{-2}\pm1.12\times10^{-2}{}^{a}$	$1.28\times10^{-2}\pm6.22\times10^{-4}{}^{a}$	$2.23\times10^{-2}\pm6.90\times10^{-3}{}^{a}$	$3.10\times10^{-2}\pm2.28\times10^{-2}{}^{a}$	$1.49\times10^{-2}\pm3.20\times10^{-3}{}^{a}$
**Alcohols**							
Mannitol	3.6886	m	27.7 ± 6.18^{a}	23.0 ± 4.72^{a}	$6.49\times10^{-1}\pm2.55\times10^{-1}{}^{b}$	17.7 ± 13.5^{a}	18.4 ± 1.86^{a}
Glycerol	3.5556	m	2.23 ± 1.17^{a}	3.99 ± 1.01^{a}	3.25 ± 1.60^{a}	6.32 ± 6.19^{a}	$4.44\pm9.58\times10^{-1}{}^{a}$
Methanol	3.3474	s	$2.37\pm3.25\times10^{-1}{}^{a}$	$1.56\pm1.19\times10^{-1}{}^{a}$	$1.36\pm1.84\times10^{-1}{}^{a}$	$1.96\pm1.21\times10^{-1}{}^{a}$	$1.69\pm2.49\times10^{-1}{}^{a}$
Ethanol	1.1706	t	90.3 ± 13.2^{b}	79.0 ± 4.85^{b}	94.1 ± 4.48^{b}	$1.34\times10^{2}\pm9.79^{a}$	$1.28\times10^{2}\pm11.9^{a}$
Propylene glycol	1.1349	d	6.04 ± 3.58^{b}	14.7 ± 2.36^{a}	$10.2\pm7.76\times10^{-1}{}^{a}$	$2.19\times10\pm8.08\times10^{-1}{}^{b}$	9.74 ± 1.53^{a}
**Ketones**							
Acetoacetate	2.2645	s	$1.07\times10^{-1}\pm1.58\times10^{-2}{}^{a}$	$1.12\times10^{-1}\pm2.43\times10^{-2}{}^{a}$	$8.32\times10^{-2}\pm2.37\times10^{-2}{}^{a}$	$1.02\times10^{-1}\pm1.53\times10^{-2}{}^{a}$	$8.96\times10^{-2}\pm1.64\times10^{-2}{}^{a}$
Succinylacetone	2.2574	s	$7.10\times10^{-2}\pm1.60\times10^{-2}{}^{a}$	$7.19\times10^{-2}\pm8.93\times10^{-3}{}^{a}$	$7.40\times10^{-2}\pm3.87\times10^{-3}{}^{a}$	$8.26\times10^{-2}\pm2.51\times10^{-3}{}^{a}$	$7.83\times10^{-2}\pm4.60\times10^{-3}{}^{a}$
Acetoin	2.2155	s	$7.57\times10^{-2}\pm9.72\times10^{-3}{}^{a}$	$6.05\times10^{-2}\pm6.07\times10^{-3}{}^{a}$	$4.84\times10^{-2}\pm8.00\times10^{-3}{}^{a}$	$8.56\times10^{-2}\pm4.32\times10^{-2}{}^{a}$	$5.73\times10^{-2}\pm8.84\times10^{-3}{}^{a}$
**Carbohydrates and Derivatives**							

myo-Inositol	3.2778	t	$8.55\times10^{-1}\pm1.49\times10^{-1}$[a]	$6.40\times10^{-1}\pm2.17\times10^{-1}$[a]	$2.57\times10^{-1}\pm4.36\times10^{-2}$[b]	$7.41\times10^{-1}\pm1.24\times10^{-1}$[a]	$7.36\times10^{-1}\pm1.01\times10^{-1}$[a]
**Nitrogenous Compounds**							
Creatine phosphate	3.9354	s	$3.11\times10^{-1}\pm4.08\times10^{-2}$[a]	$2.77\times10^{-1}\pm2.25\times10^{-2}$[a]	$2.30\times10^{-1}\pm3.92\times10^{-2}$[a]	$2.50\times10^{-1}\pm1.55\times10^{-1}$[a]	$2.75\times10^{-1}\pm2.89\times10^{-2}$[a]
Sarcosine	3.5967	s	$3.33\times10^{-1}\pm8.47\times10^{-2}$[a]	$3.13\times10^{-1}\pm1.23\times10^{-1}$[a]	$1.22\times10^{-1}\pm9.66\times10^{-2}$[a]	$4.24\times10^{-1}\pm1.71\times10^{-1}$[a]	$4.34\times10^{-1}\pm3.01\times10^{-1}$[a]
Betaine	3.2536	s	$2.18\times10^{-2}\pm9.40\times10^{-3}$[b]	$4.74\times10^{-2}\pm7.98\times10^{-3}$[b]	$2.81\times10^{-1}\pm3.55\times10^{-2}$[a]	$3.52\times10^{-2}\pm9.05\times10^{-3}$[b]	$1.55\times10^{-2}\pm5.54\times10^{-3}$[b]
O-Acetylcholine	3.2114	s	$6.40\times10^{-2}\pm2.01\times10^{-2}$[b]	$9.32\times10^{-2}\pm1.92\times10^{-2}$[b]	$1.58\times10^{-1}\pm1.11\times10^{-2}$[a]	$8.66\times10^{-2}\pm3.77\times10^{-2}$[b]	$5.67\times10^{-2}\pm1.34\times10^{-2}$[b]
Choline	3.1916	s	$2.28\times10^{-1}\pm1.66\times10^{-2}$[a]	$2.32\times10^{-1}\pm3.81\times10^{-2}$[a]	$2.25\times10^{-1}\pm2.11\times10^{-2}$[a]	$2.61\times10^{-1}\pm5.32\times10^{-2}$[a]	$2.43\times10^{-1}\pm2.72\times10^{-2}$[a]
Ethanolamine	3.1490	m	$5.43\times10^{-1}\pm1.00\times10^{-1}$[a]	$5.42\times10^{-1}\pm9.17\times10^{-2}$[a]	$4.83\times10^{-1}\pm6.24\times10^{-2}$[a]	$5.46\times10^{-1}\pm1.31\times10^{-1}$[a]	$5.26\times10^{-1}\pm8.92\times10^{-2}$[a]
Creatinine	3.0342	s	$1.15\pm7.80\times10^{-2}$[a]	$1.12\pm4.32\times10^{-2}$[a]	$9.71\times10^{-1}\pm7.50\times10^{-2}$[a]	$1.15\pm1.04\times10^{-1}$[a]	$1.02\pm1.19\times10^{-1}$[a]
Methylguanidine	2.8247	s	$2.93\times10^{-3}\pm2.47\times10^{-3}$[c]	$7.12\times10^{-2}\pm8.96\times10^{-3}$[b]	$2.08\times10^{-2}\pm9.24\times10^{-3}$[b]	$2.75\times10^{-2}\pm6.05\times10^{-3}$[b]	$8.37\times10^{-2}\pm8.36\times10^{-3}$[a]
Dimethylamine	2.7103	s	$2.97\times10^{-2}\pm2.40\times10^{-2}$[a]	$1.58\times10^{-2}\pm3.18\times10^{-4}$[a]	$1.50\times10^{-2}\pm8.54\times10^{-4}$[a]	$1.81\times10^{-2}\pm1.48\times10^{-3}$[a]	$1.57\times10^{-2}\pm1.30\times10^{-3}$[a]
**Nucleotides and Derivatives**							
Uracil	5.7972	d	$6.50\times10^{-2}\pm1.20\times10^{-2}$[b]	$1.04\times10^{-1}\pm9.67\times10^{-3}$[ab]	$1.56\times10^{-1}\pm2.16\times10^{-2}$[a]	$1.12\times10^{-1}\pm1.87\times10^{-2}$[ab]	$1.03\times10^{-1}\pm3.45\times10^{-3}$[ab]
**Phenolic and Aromatic Compounds**							
4-Hydroxyphenylacetate	7.1828	d	$9.76\times10^{-2}\pm8.62\times10^{-2}$[b]	$9.72\times10^{-2}\pm3.82\times10^{-2}$[b]	$8.34\times10^{-2}\pm1.91\times10^{-2}$[b]	$9.87\times10^{-2}\pm2.51\times10^{-2}$[b]	$2.29\times10^{-1}\pm9.77\times10^{-2}$[a]
4-Hydroxyphenyllactate	6.8420	d	$3.53\times10^{-2}\pm1.66\times10^{-2}$[b]	$5.18\times10^{-2}\pm1.23\times10^{-2}$[ab]	$6.79\times10^{-2}\pm2.07\times10^{-2}$[a]	$5.38\times10^{-2}\pm1.31\times10^{-2}$[ab]	$4.11\times10^{-2}\pm8.90\times10^{-3}$[b]

Note: Different superscript letters within the same row indicate significant differences among groups ($p < 0.05$, Kruskal-Wallis test followed by Dunn's post-hoc test with Bonferroni correction). Data are shown as mean ± SD (n=5). Chemical shift assignments are based on the Chenomx software library (Chenomx Inc., Canada, ver 8.4). s = singlet; d = doublet; t = triplet; q = quartet; m = multiplet.
